# Analyzing the spatial motion of a rigid body subjected to constant body-fixed torques and gyrostatic moment

**DOI:** 10.1038/s41598-024-55964-z

**Published:** 2024-03-05

**Authors:** T. S. Amer, H. F. El-Kafly, A. H. Elneklawy, A. A. Galal

**Affiliations:** 1 Department of Mathematics, Faculty of Science, Tanta University, Tanta, 31527 Egypt; 2 Tanta Higher Institute of Engineering and Technology, Tanta, Egypt; 3 Department of Mathematics, Faculty of Science, Kafrelsheikh University, Kafr El-Sheikh, 33516 Egypt; 4 Engineering Physics and Mathematics Department, Faculty of Engineering, Tanta University, Tanta, 31734 Egypt

**Keywords:** Nonlinear dynamics, Rigid body, Constant torques, Gyrostatic moment, Stability analysis, Applied mathematics, Aerospace engineering

## Abstract

This paper aims to explore the rotatory spatial motion of an asymmetric rigid body (RB) under constant body-fixed torques and a nonzero first component gyrostatic moment vector (GM). Euler's equations of motion are used to derive a set of dimensionless equations of motion, which are then proposed for the stability analysis of equilibrium points. Specifically, this study develops 3D phase space trajectories for three distinct scenarios; two of them are applied constant torques that are directed on the minor and major axes, while the third one is the action of applied constant torque on the body’s middle axis. Novel analytical and simulation results for both scenarios of constant torque applied along the minor and middle axes are provided in the context of separatrix surfaces, equilibrium manifolds, periodic or non-periodic solutions, and periodic solutions’ extreme. Concerning the scenario of a directed torque on the major axis, a numerical solution for the problem is presented in addition to a simulation of the graphed results for the angular velocities' trajectories in various regions. Moreover, the influence of GM is examined for each case and a full modeling for the body's stability has been present. The exceptional impact of these results is evident in the development and assessment of systems involving asymmetric RBs, such as satellites and spacecraft. It may serve as a motivating factor to explore different angles within the GM in similar cases, thereby influencing various industries, including engineering and astrophysics applications.

## Introduction

In the realm of classical mechanics, the study of gyroscopic motion is of utmost importance. One intriguing phenomenon is the gyrostatic effect, which refers to the peculiar behavior exhibited by asymmetric or symmetric RBs that are subjected to CBFTs. This effect brings fascinating dynamics and has applications in various fields such as aerospace engineering and robotics. A review of earlier research on this topic can be found in^1–20^. In^2^, the motion of an asymmetric spacecraft with a CBFT was examined. As the spacecraft spins on its minor axis and experiences energy dissipation, it becomes unstable in its orientation. Eventually, the spacecraft will realign itself to spin around its major axis. Following this maneuver, the spin rate around the major axis may be either negative or positive. In^3^, nondimensional EOMs are introduced to analyze the stability of the EPs. New analytical and simulation outcomes for CBFTs along the minor, middle, or major axes are presented. The rapid rotational motions of unbalanced asymmetric satellites around their center of mass under the influence of both gravitational and drag torques are examined in^4^. It is concluded that the satellites’ kinetic energy and angular momentum decrease, in which they are identified in the presence of Quasi stationary motion phases. Furthermore, the orbital frame of reference for the direction of angular momentum is established.

The effect of external forces and torques is investigated in^5–8^, where uniform approximate solutions are obtained using various perturbation approaches and numerical codes. These solutions are displayed to investigate the effect of the RB parameters on motion. In^9^, the author examined the movement of a single body point near a center of attraction, in which the body's space segment is comparable to an electron's orbit in a hydrogen atom. In^10^, it is looking into the viability of stabilizing a satellite's monoaxial attitude in the orbital coordinate frame through the use of an electrodynamic control system. A theorem of the asymptotic stability of body-controlled attitude motion is provided. The effectiveness of integrated controls for attitude, taking into account a distributed delay, is demonstrated through the use of numerical modeling. A previous study conducted by^11^ examined a scenario in which the rotational axis of the RB was affected by the GM and another moment around the same axis. The authors were able to achieve analytical solutions for the RB’s motion that closely aligned with the body’s physical properties, thus establishing the uniqueness of these solutions. In^12^, the issue for the RB’s movement is examined when it is subjected to a constant GM that is due to potential and gyroscopic forces. The authors successfully derived three new solutions for the EOM, which are governed by three linear invariant relationships for the angular velocity vector components. For the scenario where the RB is considered to be heavy with mass distribution, they obtained a solution that aligns with the Kovalevskaya and Goryachev-Chaplygin generalized conditions.

In^13^, the authors presented new precise solutions for the rotary movement of an RB analogous to Lagrange’s conditions. These solutions pertained to cases where the RB is subjected to a constant external torque of magnitude. Specifically, the solutions are derived for the following scenarios; firstly when the torque is parallel to the axis of symmetry and for arbitrary initial angular velocity; secondly for an orthogonal torque on this axis with stationary rotation around that axis, besides the assumption of arbitrary initial angular velocity; and finally when both the torque and initial angular velocity are perpendicular to the axis of symmetry, with the torque being fixed to the body. The kinematic solutions are represented using the rotation matrix. The obtained exact solutions are applicable to any duration of motion and rotation amplitude. In^14^, the behavior of RBs undergoing perturbed rotations near regular precession according to Lagrange’s case is investigated. The influence of a restoring moment and a slowly varying perturbing one are taken into account during the processes of the gained solutions. In the absence of resonance, an approximated system of EOM is derived for this nonlinear two-frequency system.

The required solutions are obtained in^15^ for the overall rotary movement of a nearly symmetrical RB when the action of variable torques is considered. The authors specifically focused on the scenarios of acted constant torque along the rotation’s axis, as well as variable transverse torques. In the case of RB with axial symmetry and consistent axial torque, the solutions of Euler’s EOM are completely accurate. However, the solutions of the Euler’s angles are given in approximate form. In order to consider the scenario of a rotating RB that experiences a time-varying torque in the axial direction^16^, it is necessary to expand upon the approach outlined in^15^. The resulting analytical solutions described the overall attitude movement of a near-symmetric RB that was subjected to time-varying torques around all three spatial axes relative to the body. In^17^, the analysis focused on studying the movement of an axisymmetric gyrostat satellite in a circular orbit within the impact of a Newtonian force field (NFF) is presented. It identifies and examines all the stable positions of the satellite within the orbital coordinate system, while also investigating the factors that determine their existence. Furthermore, the authors identified the specific values of the system parameters that trigger changes in the number of EPs.

In^18^, a study was conducted on the rolling of an asymmetrical RB on a horizontal plane when it is acted by a periodic GM. The authors approached the problem using a rubber body model, which assumed no slipping or spinning at the contact point. The results showed that under specific values of the system’s parameters and time-dependent of the GM, the system exhibited acceleration which resulted in an unbounded growth of energy. Further investigations were carried out to analyze how the acceleration depends on the system’s parameters and initial conditions. It should be noted that the small parameter approach has been widely utilized in^19^ as one of the approximate methods to obtain analytical solutions for the RB problem. However, the obtained periodic solutions using this approach, whether in a uniform gravity field or in an NFF; contained singular points. These singular points presented a significant challenge because the solutions aren’t defined as whole numbers or their negative counterparts. Consequently, it was crucial to address these singularities for all values of these frequencies. As a result, a significant amount of scientific research is required to bridge this gap, making it impossible to find a solution that is completely free of these singularities. In^20^, this problem was addressed by incorporating the effect of the third component of the GM, which led to the discovery of a new frequency, known as Amer's frequency. This achievement was confirmed when considering the complete impact of the GM, regardless of whether the motion of a symmetric or asymmetric RB. It was determined that the solutions obtained were free from any irregularities and were applicable for all values of this frequency.

In^21^, the author explored the analytic solution of free rotary movement of an RB that is powered by a low-power motor. Through the application of asymptotic methods, it has been shown that the motion of the carrier body is closely related to the rotation around a stationary axis, which depends on the problem's parameters and initial conditions. The analysis in^22^ focuses on investigating the equilibrium attitude and stability of a rigid spacecraft in a stationary orbit around a uniformly rotating asteroid. The linearized EOM governing attitude motion are derived based on the assumption of small motions. Subsequently, the equilibrium attitude is established for both a general and a symmetrical spacecraft. Owing to the presence of higher-order inertia integrals, the equilibrium attitude deviates slightly from zero Euler angles. In^23^, the study extends the inquiry into attitude stability to encompass a rigid spacecraft in a stationary orbit around a uniformly rotating asteroid. The authors observe that, owing to the markedly non-spherical shape and swift rotation of the asteroid, the attitude stability domain undergoes significant alterations compared to the classical stability domain predicted by the Beletskii–DeBra–Delp method for a circular orbit in a central gravity field. Notably, when the spacecraft is positioned along the intermediate-moment principal axis of the asteroid, the stability domain may exhibit a complete divergence from the classical stability expectations. In^24^, the investigation employs a differential geometric methodology to derive the Poisson tensor, Casimir functions, and equations of motion governing the phase flow and phase space structures inherent to the studied system. The equilibrium attitude of the spacecraft, serving as a stationary point for the equations of motion, is determined from a holistic perspective by considering the Hamiltonian constrained by Casimir functions. Subsequently, nonlinear stability conditions for the identified equilibrium attitude are formulated utilizing an adapted energy-Casimir method. The research further delves into the examination of nonlinear attitude stability concerning three key asteroid parameters, specifically the ratio of mean radius to orbital radius and the harmonic coefficients. In^25^, a method for depicting the turn-tensor of an axisymmetric RB using the angular momentum vector was suggested. The author proved that when specific external moments are applied, the movement of an axisymmetric RB is essentially the same as that of a spherical RB, except for the extra rotation around its axis of symmetry. Additionally, an accurate solution to the problem of unrestricted rotation of an axisymmetric RB was constructed when the impact of linear viscous friction was considered. Under the influence of the GM and NFF, an earlier investigation of the RB's problem with a zero-value assigned to the first component of the GM vector was found in^26^. To address scenarios with irrational frequencies, EOM was solved using the Poincaré method of small parameters. The influence of the GM, CBFTs, and resistive forces on a charged RB has been examined in^27^. A suitable governing system for EOM was approached using the averaging method. To reach the required results, Taylor's method was used along with some initial conditions to solve the averaged system of the EOM.

In this paper, the rotary spatial motion of an asymmetric RB with CBFTs, influenced by a first component of the GM, is investigated. To eliminate their reliance on both the inertia properties and the magnitude of torque, the controlling EOM in the case of CBFT is carried out in a dimensionless form. The determination of the dimensionless system's EPs, the derivation of the linearized EOM, the characteristic equation, and the stability features are also presented. The analytic solution for the scenario of applied CBFTs along the minor and middle axes is addressed by a comprehensive schematic simulation for the SS, trajectories, stability areas, and extreme values for the torques in the 3D phase plane. The numerical solution in the scenario of an applied CBFT along major axis is also presented, along with 3D and 2D histograms of the dimensionless angular velocities, resulting in a typical spin-up maneuver as expected in some of the analyzed regions. The impact of various GM values on the locomotion and stability trajectories is supplied because it provides a useful resource for outcomes in such a case. Finally, each scenario includes a detailed examination of the minimum and maximum values of different dimensionless angular velocity components.

## Problem’s formulation

**Figure 1:**
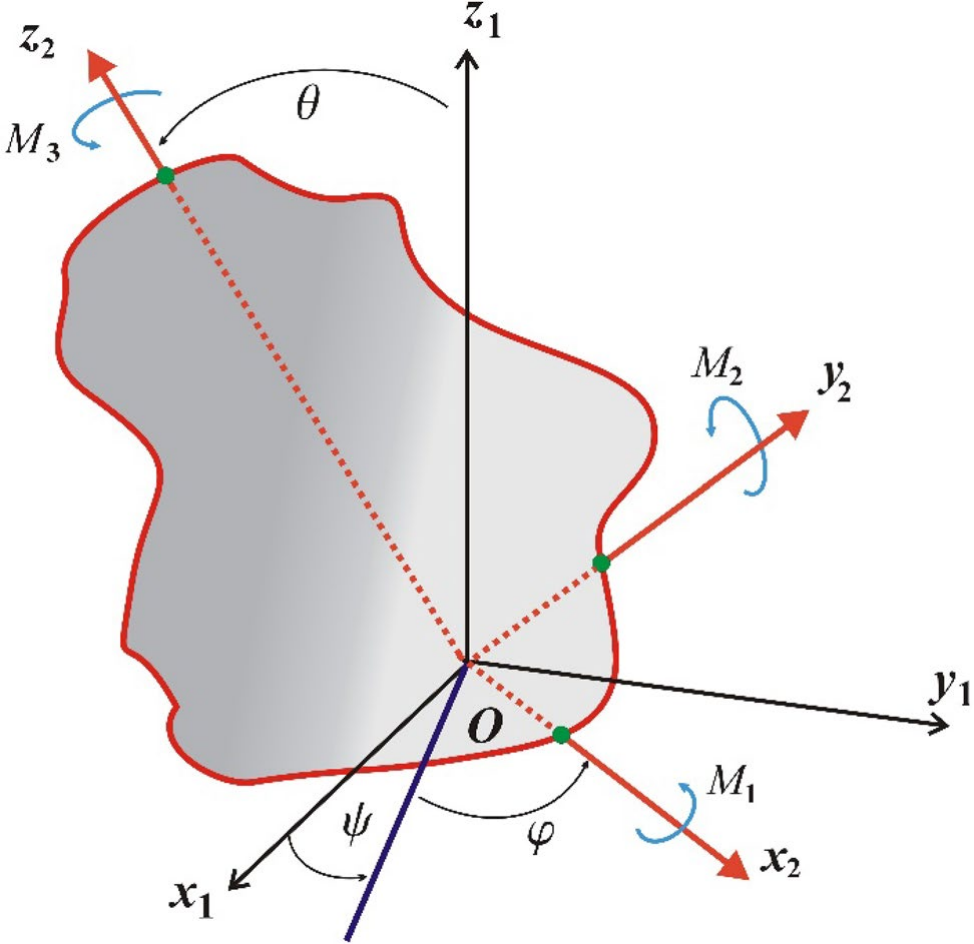
The simulation model of the RB's motion.

In order to improve the description of this problem, this section seeks to provide us with more information. Therefore, the spatial rotation of an asymmetric RB about a fixed origin $O$ of two coordinate systems is considered. The first coordinate system $Ox_{1} y_{1} z_{1}$ is the inertial and the second system $Ox_{2} y_{2} z_{2}$ rotates with the body. The RB's motion is influenced by both GM $\underline {\lambda }$ and CBFTs $\underline {M}$ vectors, as graphed in Fig. 1.

The governing EOM for the body^1,6,27^, is given by

1 $$\begin{aligned} & D_{1} \frac{{d\omega_{1} }}{dt} + (D_{3} - D_{2} )\omega_{2} \omega_{3} = M_{1} , \\ & D_{2} \frac{{d\omega_{2} }}{dt} + (D_{1} - D_{3} )\omega_{3} \omega_{1} + \lambda_{1} \omega_{3} = M_{2} , \\ & D_{3} \frac{{d\omega_{3} }}{dt} + (D_{2} - D_{1} )\omega_{1} \omega_{2} - \lambda_{1} \omega_{2} = M_{3} . \\ \end{aligned}$$

Here, $D = (D_{1} ,D_{2} ,D_{3} )$ is the tensor components of the principal inertia’s moment such as $D_{1} > D_{2} > D_{3}$, $\underline {\omega } = (\omega_{1} ,\omega_{2} ,\omega_{3} )$ represents the body's angular velocity, and $(M_{1} ,M_{2} ,M_{3} )$ are the components of the CBFTs $\underline{M}$, while $\lambda_{1}$ is the first component of the GM $\underline {\lambda }$ (where $\lambda_{2} = \lambda_{3} = 0$) along $Ox_{2}$, and $t$ represents the time. Taking into consideration the following new parameters to eliminate the dependence of the above system on the inertial properties of the RB as

2 $$\begin{aligned} & k_{1} = {{(D_{2} - D_{3} )} \mathord{\left/ {\vphantom {{(D_{2} - D_{3} )} {D_{1} }}} \right. \kern-0pt} {D_{1} }},\,\,\,\,\,\,\,\,k_{2} = {{(D_{1} - D_{3} )} \mathord{\left/ {\vphantom {{(D_{1} - D_{3} )} {D_{2} }}} \right. \kern-0pt} {D_{2} }},\,\,\,\,\,\,\,\,k_{3} = {{(D_{1} - D_{2} )} \mathord{\left/ {\vphantom {{(D_{1} - D_{2} )} {D_{3} }}} \right. \kern-0pt} {D_{3} }},\, \\ & k = k_{1} k_{2} k_{3} ,\,\,\,\,\,\,\,\,\tau = \sqrt k \,t,\,\,\,\,\,\,\,\,a = {{\lambda_{1} } \mathord{\left/ {\vphantom {{\lambda_{1} } {(D_{2} k_{2} \sqrt {k_{1} } }}} \right. \kern-0pt} {(D_{2} k_{2} \sqrt {k_{1} } }}),\,\,\,\,\,\,\,\,b = {{\lambda_{1} } \mathord{\left/ {\vphantom {{\lambda_{1} } {(D_{3} k_{3} \sqrt {k_{1} } }}} \right. \kern-0pt} {(D_{3} k_{3} \sqrt {k_{1} } }}), \\ & u_{j} = {{M_{j} } \mathord{\left/ {\vphantom {{M_{j} } {D_{j} }}} \right. \kern-0pt} {D_{j} }},\,\,\,\,\,\,\,\,\,\mu_{j} = {{u_{j} } \mathord{\left/ {\vphantom {{u_{j} } {(\sqrt k \sqrt {k_{j} } )}}} \right. \kern-0pt} {(\sqrt k \sqrt {k_{j} } )}},\,\,\,\,\,\,\,\,x_{j} = {{\omega_{j} } \mathord{\left/ {\vphantom {{\omega_{j} } {\sqrt {k_{j} } }}} \right. \kern-0pt} {\sqrt {k_{j} } }},\,\,\,\,\,\,\,\,(j = 1,2,3). \\ \end{aligned}$$

Therefore, one can rewrite the equation of system (1) as follows

3 $$\begin{aligned} & \frac{{dx_{1} }}{d\tau } - x_{2} x_{3} = \mu_{1} , \\ & \frac{{dx_{2} }}{d\tau } + x_{3} x_{1} + ax_{3} = \mu_{2} , \\ & \frac{{dx_{3} }}{d\tau } - x_{1} x_{2} - bx_{2} = \mu_{3} , \\ \end{aligned}$$

where the scaled quantities $x_{j}$, $\mu_{j}$, and $\tau$ represent the angular velocities, CBFTs, and time, respectively.

The system in (1) can no longer rely on the magnitude of the torque vector $(M_{1} ,M_{2} ,M_{3} )$ as $u = {{\sqrt {k_{2} k_{3} u_{1}^{2} + k_{3} k_{1} u_{2}^{2} + k_{1} k_{2} u_{3}^{2} } } \mathord{\left/ {\vphantom {{\sqrt {k_{2} k_{3} u_{1}^{2} + k_{3} k_{1} u_{2}^{2} + k_{1} k_{2} u_{3}^{2} } } k}} \right. \kern-0pt} k}$ is introduced. Making good use of $u$ to redefine $x_{j}$, $\mu_{j}$, and $\tau$ as follows

4 $$\tau = \sqrt {k{\kern 1pt} u} \,t,\,\,\,\,\,\,\,\,x_{j} = {{\omega_{j} } \mathord{\left/ {\vphantom {{\omega_{j} } {\sqrt {u{\kern 1pt} k_{j} } ,}}} \right. \kern-0pt} {\sqrt {u{\kern 1pt} k_{j} } ,}}\,\,\,\,\,\,\,\,\mu_{j} = ({1 \mathord{\left/ {\vphantom {1 {u{\kern 1pt} \sqrt k }}} \right. \kern-0pt} {u{\kern 1pt} \sqrt k }}){{(u_{j} } \mathord{\left/ {\vphantom {{(u_{j} } {\sqrt {k_{j} } ).}}} \right. \kern-0pt} {\sqrt {k_{j} } ).}}$$

Substituting (4) into system (1) to yield

5 $$\begin{aligned} & \frac{{dx_{1} }}{d\tau } - x_{2} x_{3} = \mu_{1} , \\ & \frac{{dx_{2} }}{d\tau } + x_{3} x_{1} + ax_{3} = \mu_{2} , \\ & \frac{{dx_{3} }}{d\tau } - x_{1} x_{2} - bx_{2} = \mu_{3} , \\ \end{aligned}$$

where the adjusted quantities $x_{j} ,\,\,\mu_{j} ,$ and $\tau$ represent, respectively, the dimensionless forms of the angular velocities, CBFTs, and time. According to system (4), one gets

6 $$\mu_{1}^{2} + \mu_{2}^{2} + \mu_{3}^{2} = 1.$$

Therefore, the EOM at a steady state becomes

7 $$\begin{aligned} & X_{2} X_{3} = - \mu_{1} , \\ & X_{3} X_{1} + aX_{3} = \mu_{2} , \\ & X_{1} X_{2} + bX_{2} = - \mu_{3} , \\ \end{aligned}$$

where $X_{j}$ are the components of the EPs. Therefore, one obtains

8 $$X_{2}^{2} X_{3}^{2} (X_{1} + a)(X_{1} + b) = \mu_{1} \mu_{2} \mu_{3} .$$

In other words, if and only if $\mu_{1} \,\mu_{2} \mu_{3} \ge 0$, the EPs exist. Eight EPs could be determined by knowing the value of the constant torque $\mu_{j}$ as follows

9 $$\begin{aligned} (X_{1} + a)(X_{1} + b) & = {{\mu_{2} \mu_{3} } \mathord{\left/ {\vphantom {{\mu_{2} \mu_{3} } {\mu_{1} }}} \right. \kern-0pt} {\mu_{1} }}, \\ X_{2} & = \pm \sqrt {\frac{{\mu_{3} \mu_{1} (X_{1} + a)}}{{\mu_{2} (X_{1} + b)}}} , \\ X_{3} & = \pm \sqrt {\frac{{\mu_{1} \mu_{2} (X_{1} + b)}}{{\mu_{3} (X_{1} + a)}}} . \\ \end{aligned}$$

Let us define the state perturbation concepts of angular velocity as^3^

10 $$\Delta {\kern 1pt} x_{j} = x_{j} - X_{j} .$$

Then taking into account the consistency of $\mu_{j}$, to derive the below linearized EOM

11 $$\left. {\left( {\begin{array}{*{20}c} {\Delta \dot{x}_{1} } \\ {\Delta \dot{x}_{2} } \\ {\Delta \dot{x}_{3} } \\ \end{array} } \right.} \right) = \left. {\left( {\begin{array}{*{20}c} 0 & {X_{3} } & {X_{2} } \\ { - X_{3} } & 0 & { - (X_{1} + a)} \\ {X_{2} } & {(X_{1} + b)} & 0 \\ \end{array} } \right.} \right)\left. {\left( {\begin{array}{*{20}c} {\Delta x_{1} } \\ {\Delta x_{2} } \\ {\Delta x_{3} } \\ \end{array} } \right.} \right).$$

Then, the characteristic equation for (11) can thus be expressed as

12 $$\zeta^{3} + \zeta [(X_{1} + a)(X_{1} + b) - X_{2}^{2} + X_{3}^{2} ] + X_{2} X_{3} (2X_{1} + a + b) = 0.$$

The prerequisites for the existence and stability of the roots of Eq. (12) are given in Table 1 along with an extensive list of all achievable and possible combinations of CBFTs and angular velocities.

**Table 1:** Explores the linear stability of different EPs.

Case	EP	Characteristic equation	Stability Manner	CBFT	Notes
1	$(0,0,0)$	$\zeta^{3} = 0$	Unstable	$(0,0,0)$	None
2	$(X_{1} ,0,0)$	$\zeta^{3} + \zeta [(X_{1} + a) \times (X_{1} + b)] = 0$	Stable	$(0,0,0)$	None
3	$(0,X_{2} ,0)$	$\zeta^{3} - \zeta X_{2}^{2} = 0$	Unstable	$(0,0,0)$	None
4	$(0,0,X_{3} )$	$\zeta^{3} + \zeta X_{3}^{2} = 0$	Stable	$(0,0,0)$	None
5	$(0,X_{2} ,X_{3} )$	$\zeta^{3} - \zeta (X_{2}^{2} - X_{3}^{2} ) + X_{2} X_{3} (a + b) = 0$	Stable if $\left| {X_{2} } \right| < \left| {X_{3} } \right|$	$(\mu_{1} ,0,0)$	$X_{2} X_{3} = - \mu_{1}$
6	$(X_{1} ,0,X_{3} )$	$\zeta^{3} + \zeta [(X_{1} + a)(X_{1} + b) + X_{3}^{2} ] = 0$	Stable if $X_{1} \ne - a$	$(0,\mu_{2} ,0)$	$X_{3} = {{\mu_{2} } \mathord{\left/ {\vphantom {{\mu_{2} } {(X_{1} + a)}}} \right. \kern-0pt} {(X_{1} + a)}}$
7	$(X_{1} ,X_{2} ,0)$	$\zeta^{3} + \zeta [(X_{1} + a)(X_{1} + b) - X_{2}^{2} ] = 0$	Stable if $X_{1} \ne - b$	$(0,0,\mu_{3} )$	$X_{2} = {{ - \mu_{3} } \mathord{\left/ {\vphantom {{ - \mu_{3} } {(X_{1} + b)}}} \right. \kern-0pt} {(X_{1} + b)}}$
8	$(X_{1} ,X_{2} ,X_{3} )$	$$\begin{gathered} \zeta^{3} + \zeta [(X_{1} + a)(X_{1} + b) - X_{2}^{2} + X_{3}^{2} ] \hfill \\ \,\,\,\,\,\, + X_{2} X_{3} (2X_{1} + a + b) = 0. \hfill \\ \end{gathered}$$	Unstable	$(\mu_{1} ,\mu_{2} ,\mu_{3} )$	$\mu_{1} \mu_{2} \mu_{3} > 0,\,\,\left\| \mu \right\| = 1$

## CBFT along the minor axis

In this section, an analytical approach that determines the angular velocities of the RB when undergoing CBFT along its minor axis is investigated. Therefore, at the value $(\mu_{1} ,\mu_{2} ,\mu_{3} ) = (0,0,1)$ along this axis, system (5) takes the form

13 $$\begin{aligned} \frac{{dx_{1} }}{d\tau } - x_{2} x_{3} & = 0, \\ \frac{{dx_{2} }}{d\tau } + x_{3} x_{1} + ax_{3} & = 0, \\ \frac{{dx_{3} }}{d\tau } - x_{1} x_{2} - bx_{2} & = 1. \\ \end{aligned}$$

These equations depict the EPs of the system, which form a characterized hyperbola according to the equation $X_{2} (X_{1} + b) = - 1$. Additionally, they are stable at $\left| {X_{2} } \right| > 1$ and unstable at $\left| {X_{1} + b} \right| > 1$. According to^1^, a new variable $\alpha_{1}$ can be inserted as

14 $$\alpha_{1} (\tau ) = \int\limits_{0}^{\tau } {x_{3} (\sigma_{1} )\,d\sigma_{1} } ,$$

which also can be rewritten as ${{d\alpha_{1} } \mathord{\left/ {\vphantom {{d\alpha_{1} } {d\tau }}} \right. \kern-0pt} {d\tau }} = x_{3} (\tau );\,\,\,\alpha_{1} (0) = 0$, and then one can transform system (13) into

15 $$\begin{aligned} \frac{{dx_{1} }}{{d\alpha_{1} }} - x_{2} & = 0, \\ \frac{{dx_{2} }}{{d\alpha_{1} }} + x_{1} + a & = 0, \\ \frac{{d^{2} \alpha_{1} }}{{d\tau^{2} }} - x_{1} x_{2} - bx_{2} & = 1. \\ \end{aligned}$$

By taking the derivative of the second equation from (15) with respect to $\alpha_{1}$, and subsequently employing the first equation to get

16 $$\frac{{d^{2} x_{2} }}{{d\alpha_{1}^{2} }} + x_{2} = 0.$$

which is a simple harmonic motion equation. Therefore, the following solutions hold for the first two equations of system (15)

17 $$x_{1} = F\sin (\alpha_{1} + \psi_{1} ),\,\,\,x_{2} = F\cos (\alpha_{1} + \psi_{1} ).$$

where $F = \sqrt {x_{1}^{2} (0) + x_{2}^{2} (0)}$ and $\psi_{1} = \tan^{ - 1} {{[x_{1} (0)} \mathord{\left/ {\vphantom {{[x_{1} (0)} {x_{2} (0)}}} \right. \kern-0pt} {x_{2} (0)}}]$ as $\left| {\psi_{1} } \right| \le \pi$. Therefore, one writes

18 $$x_{1}^{2} + x_{2}^{2} = F^{2} .$$

This circle's radius $F$ is determined solely by the initial values respected to dimensionless first and second angular velocity components, which represents the dimensionless first integral of the motion. Substituting Eq. (17) into the third equation in system (15), yields

19 $$\frac{{d^{2} \alpha_{1} }}{{d\tau^{2} }} - F\left[ {\frac{F}{2}\sin 2(\alpha_{1} + \psi_{1} ) - b\cos (\alpha_{1} + \psi_{1} )} \right] = 1.$$

Let's assume that

20 $$\alpha = 2(\alpha_{1} + \psi_{1} ).$$

As a consequence, Eq. (18) undergoes reformulation as follows

21 $$\frac{{d^{2} \alpha }}{{d\tau^{2} }} - F\left( {F\sin \alpha - 2b\cos \frac{\alpha }{2}} \right) = 2.$$

At the equilibrium's angle $\alpha^{ * }$ with the absence of the GM, $\sin \alpha^{ * } = {{ - 2} \mathord{\left/ {\vphantom {{ - 2} {F^{2} }}} \right. \kern-0pt} {F^{2} }}$ is satisfied. Evidently, there is no EP for $F < \sqrt 2$. To investigate the system's stability properties in the scenario where $F \ge \sqrt 2$ will be presented. Therefore, the following new parameter is introduced in the form

22 $$x = \frac{d\alpha }{{d\tau }}.$$

This means that Eq. (21) has been transformed in the $(\alpha ,x)$ phase plane as

23 $$\frac{dx}{{d\tau }} = F\left( {F\sin \alpha - 2b\cos \frac{\alpha }{2}} \right) + 2.$$

Based on Eqs. (22) and (23), one can write

24 $$\frac{dx}{{d\alpha }} = \frac{{F\,[F\sin \alpha - 2b\cos ({\alpha \mathord{\left/ {\vphantom {\alpha 2}} \right. \kern-0pt} 2})] + 2}}{x}.$$

**Figure 2:**
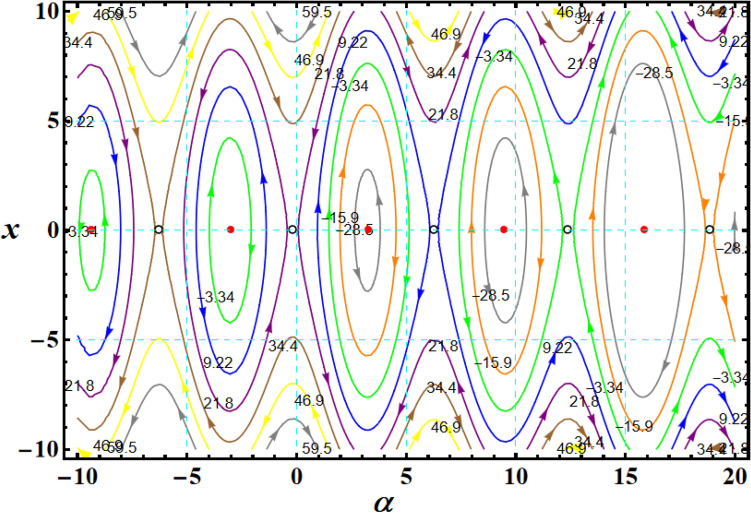
Shows trajectories for CBFT along minor axis at $x(0) = (2.5,4,0.1),$ $D( = 2000,1500,1000)\;{\text{kg}}\;{\text{m}}^{2} ,$ and $\lambda_{1} = 100\;{\text{kg}}\;m^{2} \;s^{ - 1}$.

Likewise noticing that for a certain trajectory, the motion's first integral $F$ is constant. Therefore, Eq. (24) can be integrated to get

25 $$\frac{{x^{2} }}{2} + F^{2} \cos \alpha - 4Fb\sin \frac{\alpha }{2} - 2\alpha = G_{1} ,$$

where the second integral of the motion corresponds to the dimensionless energy constant $G_{1}$. It may be contour-plotted on the $(\alpha ,x)$ phase plane using Eq. (25) and provided a specific value for the dimensionless first integral of the motion $F$. This type of contour-plot is shown in Fig. 2 for the value $F = 4.72$, where each dashed or solid line contour corresponds to a $G_{1}$ trajectory with constant values. The phase plane axis is periodically positioned along unlimited stable centers ($\bullet$) and unstable saddles ($\circ$) when $F \ge 1.41$.

Therefore, $(\alpha^{ * } ,x^{ * } ) = [\sin^{ - 1} ({{ - 2} \mathord{\left/ {\vphantom {{ - 2} {F^{2} }}} \right. \kern-0pt} {F^{2} }}),0]$, where $\left| {\sin^{ - 1} \varphi_{1} } \right| \le {\pi \mathord{\left/ {\vphantom {\pi 2}} \right. \kern-0pt} 2}$ for all $\varphi_{1}$, is the first EP inside $(\alpha ,x)$ concerning left of the origin. Center points (CPs) are situated at $\pi - \alpha^{ * } \pm 2m\pi \,;\,\,\,\,(m = 1,2, \ldots ),$ while the saddle points (SPs) are at $\alpha^{ * } \pm 2m\pi$. For an SP $\alpha_{s}$, the energy constant $G_{1s}$ takes the form as

26 $$G_{1s} = F^{2} \cos \alpha_{s} - 4Fb\sin ({{\alpha_{s} } \mathord{\left/ {\vphantom {{\alpha_{s} } 2}} \right. \kern-0pt} 2}) - 2\alpha_{s} .$$

Moreover, the separatrix's equation travels via $\alpha_{s}$ is

27 $$\frac{{x^{2} }}{2} + F^{2} \cos \alpha - 4Fb\sin \frac{\alpha }{2} - 2\alpha = G_{1s} .$$

Substituting (26) into (27) yields

28 $$x = \pm \sqrt 2 F\sqrt {(\cos \alpha_{s} - \cos \alpha ) - 4b\{ [{{\sin ({{\alpha_{s} } \mathord{\left/ {\vphantom {{\alpha_{s} } {2)}}} \right. \kern-0pt} {2)}} - \sin ({\alpha \mathord{\left/ {\vphantom {\alpha {2)}}} \right. \kern-0pt} {2)}}]} \mathord{\left/ {\vphantom {{\sin ({{\alpha_{s} } \mathord{\left/ {\vphantom {{\alpha_{s} } {2)}}} \right. \kern-0pt} {2)}} - \sin ({\alpha \mathord{\left/ {\vphantom {\alpha {2)}}} \right. \kern-0pt} {2)}}]} {F\} }}} \right. \kern-0pt} {F\} }} + (\alpha_{s} - \alpha )\sin \alpha_{s} } \,.$$

**Figure 3:**
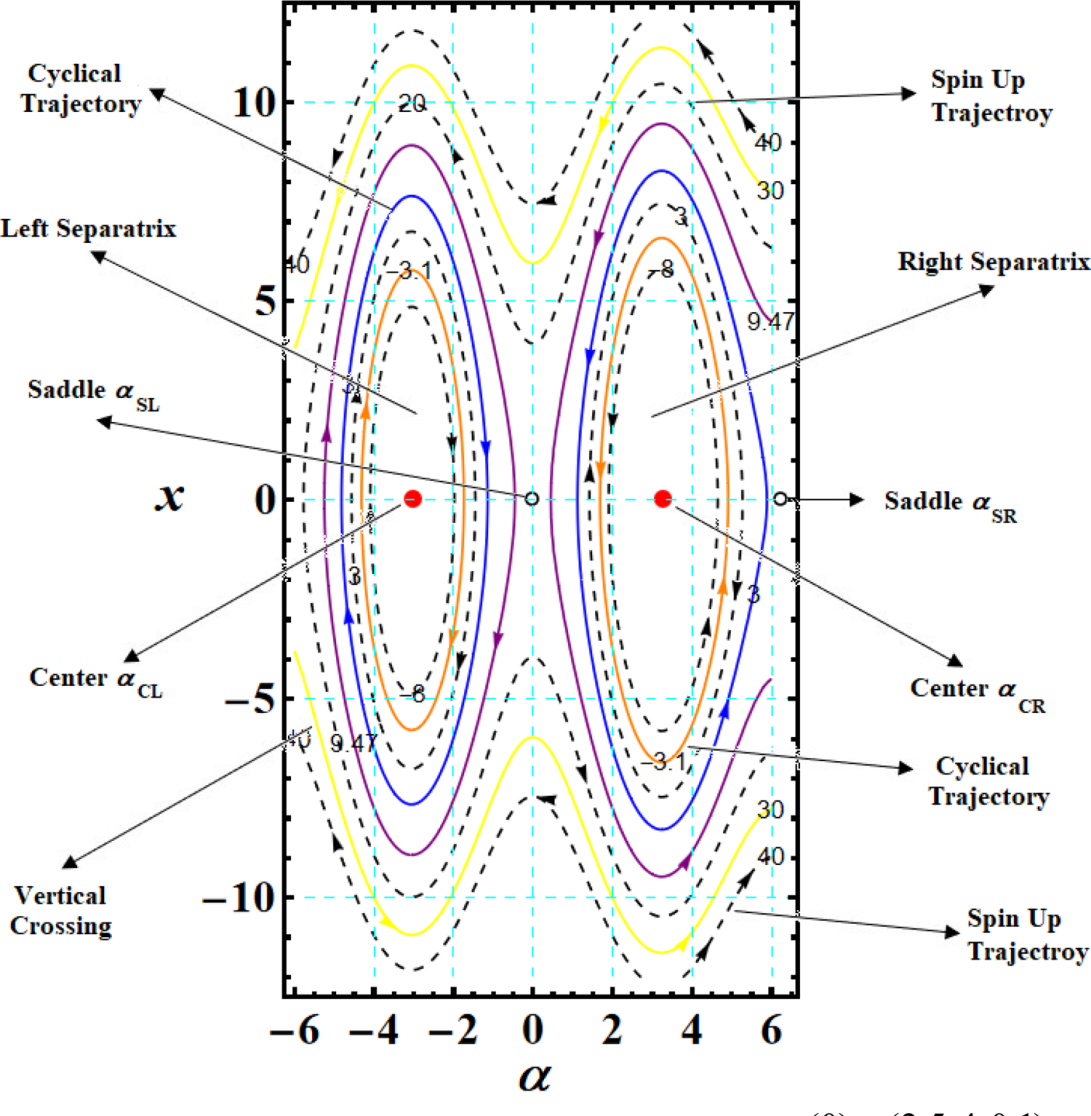
Plots LS and RS for a constant minor torque axis at $$x(0) = (2.5,4,0.1),$$$$D( = 2000,1500,1000)\;{\text{kg}}\;{\text{m}}^{2} ,$$ and $$\lambda_{1} = 100\;{\text{kg}}\;{\text{m}}^{2} \;{\text{s}}^{ - 1}$$.

Bringing back that $\alpha (0) = 2\psi_{1}$ and $\left| {\psi_{1} } \right| \le \pi$. Regarding stability analysis, there are just two separatrices that are relevant: the left separatrix (LS), going via the left SP at $\alpha_{SL}$ and wrapping around the left CP at $\alpha_{CL}$; the right separatrix (RS), going via the right SP at $\alpha_{SR}$ and wrapping around the right CP at $\alpha_{CR}$. The LS and RS at $F = 4.72$ are displayed in Fig. 3. The various CPs and SPs connected to the LS and RS are also represented. Additionally, it illustrates the cyclical, spin-up, and vertical crossing (VC) trajectories throughout the action.

**Figure 4:**
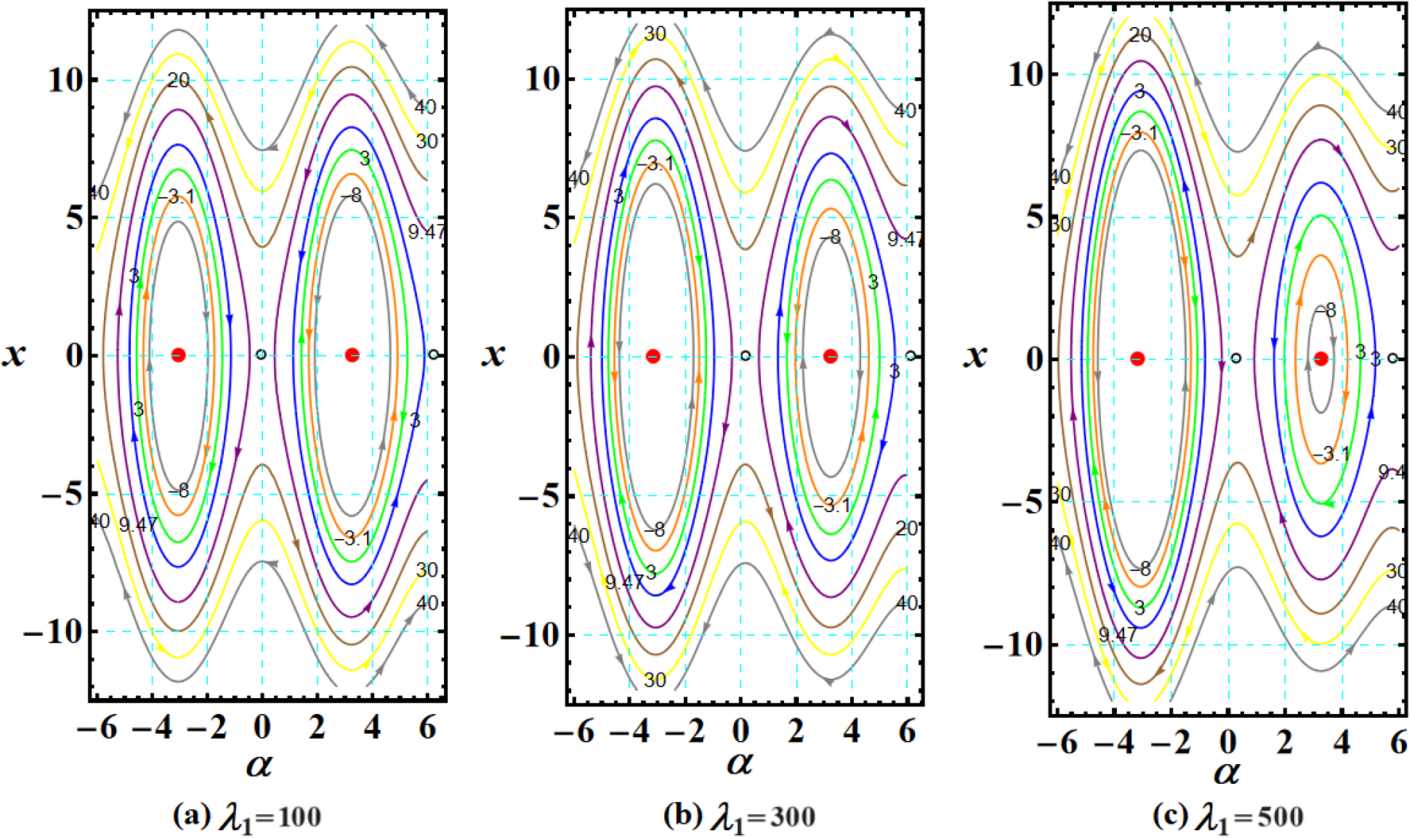
Plots of LS and RS for a constant minor torque axis at $x(0) = (2.5,4,0.1)$, $D( = 2000,1500,1000)\;{\text{kg}}\;{\text{m}}^{2}$, and $\lambda_{1} ( = 100,300,500)\;{\text{kg}}\;{\text{m}}^{2} \;{\text{s}}^{ - 1}$.

The graphed curves in Fig. 4 expresses the impact of distinct GM’s values $\lambda_{1} ( = 100,300,500)\,{\text{kg}}\,{\text{m}}^{2}\,{\text{s}}^{ - 1}$ that acted on the body’s main axes of inertia. The distinction between every case has been shown in the graphical simulation as in (a) of Fig. 4 which represents the LS and RS at $\lambda_{1} = 100\,{\text{kg}}\,{\text{m}}^{2}\,{\text{s}}^{ - 1}$, noticing that the cyclical trajectories at the RS make closed oval trajectories bigger than the LS with a united direction for the VC and the spin-up trajectories. The left SP $\alpha_{SL}$ and right one $\alpha_{SR}$ are located, respectively, at $(0,0)$ and $(6.2,0)$, while the left CP $\alpha_{CL}$ and the right one $\alpha_{CR}$ are found, respectively, at $( - 3,0)$ and $(3.3,0)$ on the $(\alpha ,x)$ phase plane. At $\lambda_{1} = 300\,{\text{kg}}\,{\text{m}}^{2}\,{\text{s}}^{ - 1}$, as seen in Fig. 4b, the cyclical trajectories at the RS make distinct closed ovals other than the aforementioned at $\lambda_{1} = 100$, which are now smaller than the LS, with a non-united direction for the VC and the spin-up trajectories. In this case, the left SP is located at $(0.19,0)$ and the position of the right SP is found at $(6.15,0)$, while the left CP is found at $( - 3.1,0)$ and the location of the right CP is found at $(3.29,0)$. When the value of the GM becomes $\lambda_{1} = 500\,{\text{kg}}\,{\text{m}}^{2}\,{\text{s}}^{ - 1}$, the difference between the two SS becomes so obvious as the LS is becoming much smaller than the RS with a distribution in the direction of the trajectories, see Fig. 4c. The left SP is now located at $(0.32,0)$ and the right one SP is found at $(5.8,0)$, while the left CP and the right CP are located at $( - 3.13,0)$ and $(3.28,0)$, respectively.

Considering

29 $$x = 2x_{3} ,\,\,\,\,\,\,\cos \alpha = \frac{{1 - \tan^{2} ({\alpha \mathord{\left/ {\vphantom {\alpha 2}} \right. \kern-0pt} 2})}}{{1 + \tan^{2} ({\alpha \mathord{\left/ {\vphantom {\alpha 2}} \right. \kern-0pt} 2})}} = \frac{{x_{2}^{2} - x_{1}^{2} }}{{F^{2} }};\,\,\,\,\,\,\alpha = 2\tan^{ - 1} {{(x_{1} } \mathord{\left/ {\vphantom {{(x_{1} } {x_{2} )}}} \right. \kern-0pt} {x_{2} )}}.$$

The substitution from (29) into (27), yields

30 $$2x_{3}^{2} + x_{2}^{2} - x_{1}^{2} - 4bx_{1} - 4\tan^{ - 1} ({{x_{1} } \mathord{\left/ {\vphantom {{x_{1} } {x_{2} }}} \right. \kern-0pt} {x_{2} }}) = G_{1} .$$

It must be noted that $\cos \alpha_{s} = {{\sqrt {(F^{4} - 4)} } \mathord{\left/ {\vphantom {{\sqrt {(F^{4} - 4)} } {A^{2} }}} \right. \kern-0pt} {A^{2} }}$ and $\sin ({{\alpha_{s} } \mathord{\left/ {\vphantom {{\alpha_{s} } 2}} \right. \kern-0pt} 2}) = \pm \sqrt {{{(1 - \cos \alpha_{s} )} \mathord{\left/ {\vphantom {{(1 - \cos \alpha_{s} )} 2}} \right. \kern-0pt} 2}}$. Therefore, the substitution from (26) into (30), yields

31 $$\begin{gathered} 2x_{3}^{2} + x_{2}^{2} - x_{1}^{2} - 4bx_{1} - \sqrt {(x_{1}^{2} + x_{2}^{2} )^{2} - 4} - 2\sin^{ - 1} [{2 \mathord{\left/ {\vphantom {2 {(x_{1}^{2} + x_{2}^{2} }}} \right. \kern-0pt} {(x_{1}^{2} + x_{2}^{2} }})] - 4\tan^{ - 1} ({{x_{1} } \mathord{\left/ {\vphantom {{x_{1} } {x_{2} }}} \right. \kern-0pt} {x_{2} }}) \hfill \\ \,\,\,\,\,\,\,\,\, \pm ({{4b} \mathord{\left/ {\vphantom {{4b} {\sqrt 2 }}} \right. \kern-0pt} {\sqrt 2 }})\sqrt {x_{1}^{2} + x_{2}^{2} - \sqrt {(x_{1}^{2} + x_{2}^{2} )^{2} - 4} } = 0, \hfill \\ \end{gathered}$$

**Figure 5:**
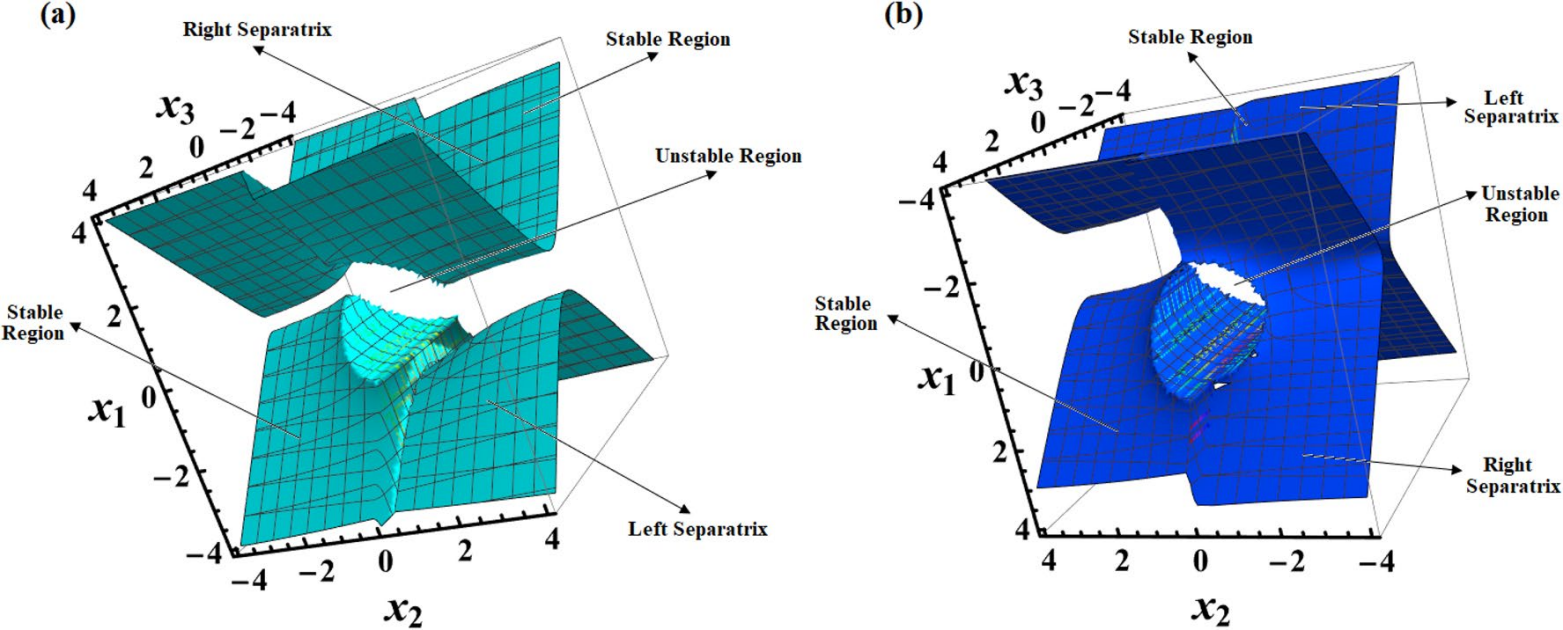
Shows the 3D simulation for LS and RS for a constant minor torque axis when $D( = 2000,1500,1000){\text{kg}}{\text{.m}}^{2}$, and $\lambda_{1} = 100{\text{kg}}\;{\text{m}}^{2} \;{\text{s}}^{ - 1}$.

Parts of Fig. 5 show 3D representations of the LS and RS surfaces at $D( = 2000,1500,1000)\,{\text{kg}}\,{\text{m}}^{2}$ when $\lambda_{1} = 100\,{\text{kg}}\,{\text{m}}^{2}\,{\text{s}}^{ - 1}$ in the space $(x_{1} ,x_{2} ,x_{3} )$. It is noted that the mentioned space is divided into three infinite areas by two surfaces. Two of them are stable, while the third one is an unstable region. One of these areas will remain associated with any motion that has starting conditions there. If the body starts its motion within one of the stable domains, it will follow a closed and limited path within that region, revolving around its respective center. However, if the initial conditions lie within the realm of instability, an upward spin will be executed. When these conditions lie in the unstable region, the projection of the endpoint dimensionless angular velocity $(x_{1} ,x_{2} ,x_{3} )$ inside $(x_{1} ,x_{2} )$ plane represents a complete circle, and for initial conditions within any of the stable regions, it represents a segment of the circle. The circle's radius, denoted as $F$, has the flexibility to assume any length, leading to the possibility of both stable and unstable movement.

Figure 5 displays a contour map of the top area $(x_{1} ,x_{2} ,x_{3} > 0)$ for the LS and RS seen in Fig. 3. The EPs for the system are found on the two branches of the hyperbola $x_{2} (x_{1} + b) = - 1$. The equation $x_{1} + x_{2} = 0$ serves as a dividing line, creating distinct regions within the hyperbola branches known as Lyapunov stable and unstable domains, as shown in the cylindrical coordinates $F = \sqrt {x_{1}^{2} (0) + x_{2}^{2} (0)}$ and $\psi_{1} = \tan^{ - 1} {{[x_{1} (0)} \mathord{\left/ {\vphantom {{[x_{1} (0)} {x_{2} (0)}}} \right. \kern-0pt} {x_{2} (0)}}]$, which are presented. Within the circumference of a circle with a radius of $F$, the absence of EPs can be observed.

**Figure 6:**
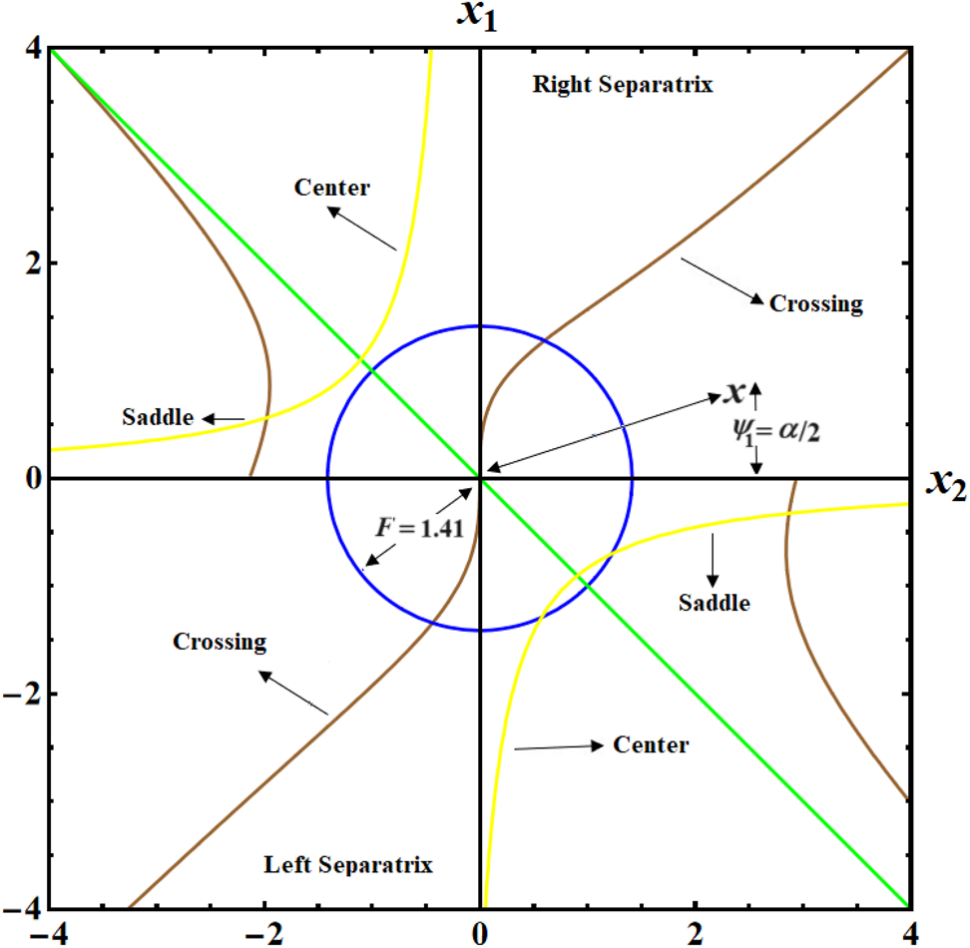
Shows contour plots of LS and RS surfaces for a constant minor torque axis.

Figure 6 shows the depiction, at a moment of principal inertia $D( = 2000,1500,1000){\text{kg}}{\text{.m}}^{2}$, the initial condition $x(0) = (2.5,4,0.1)$, and the value of the GM $\lambda_{1} = 100{\text{kg}}{\text{.m}}^{2} {\text{.s}}^{ - 1}$, for the following surfaces:

1. the circle $(x_{1}^{2} + x_{2}^{2} )^{2} - 4 = 0$ where none of the EPs are located,
2. the two branches of the hyperbola $x_{2} (x_{1} + b) = - 1$, which are where all of the system's EPs are located,
3. the line $x_{1} + x_{2} = 0$ that separates the two branches of the hyperbola $x_{2} (x_{1} + b) = - 1$ into regions with Lyapunov stable and unstable regions,
4. the surface $$x_{2}^{2} - x_{1}^{2} - 4bx_{1} - 4\tan^{ - 1} ({{x_{1} } \mathord{\left/ {\vphantom {{x_{1} } {x_{2} }}} \right. \kern-0pt} {x_{2} }}) = 0$$.

The SP, CP, and VC points connected to the LS and RS in Fig. 2 have the same positions where a circle of radius $F = 1.41$ intersects with the SP, CP, and VC curves in Fig. 6.

In order to find the solutions to the transcendental equations provided by Eq. (28), one must find the intersection of the two SS and the $(x_{1} ,x_{2} )$ plane as follows

32 $$(\cos \alpha_{s} - \cos \alpha ) - 4b\{ [{{\sin ({{\alpha_{s} } \mathord{\left/ {\vphantom {{\alpha_{s} } {2)}}} \right. \kern-0pt} {2)}} - \sin ({\alpha \mathord{\left/ {\vphantom {\alpha {2)}}} \right. \kern-0pt} {2)}}]} \mathord{\left/ {\vphantom {{\sin ({{\alpha_{s} } \mathord{\left/ {\vphantom {{\alpha_{s} } {2)}}} \right. \kern-0pt} {2)}} - \sin ({\alpha \mathord{\left/ {\vphantom {\alpha {2)}}} \right. \kern-0pt} {2)}}]} F}} \right. \kern-0pt} F}\} + (\alpha_{s} - \alpha )\sin \alpha_{s} = 0\,.$$

The fact that $\alpha = \alpha_{s}$ is thought to be a solution of (29), which results in

33 $$\sin \alpha_{s} = \sin \alpha = \frac{{2\tan {{(\alpha } \mathord{\left/ {\vphantom {{(\alpha } {2)}}} \right. \kern-0pt} {2)}}}}{{1 + \tan^{2} {{(\alpha } \mathord{\left/ {\vphantom {{(\alpha } {2)}}} \right. \kern-0pt} {2)}}}} = \frac{{2x_{1} x_{2} }}{{x_{1}^{2} + x_{2}^{2} }}.$$

**Figure 7:**
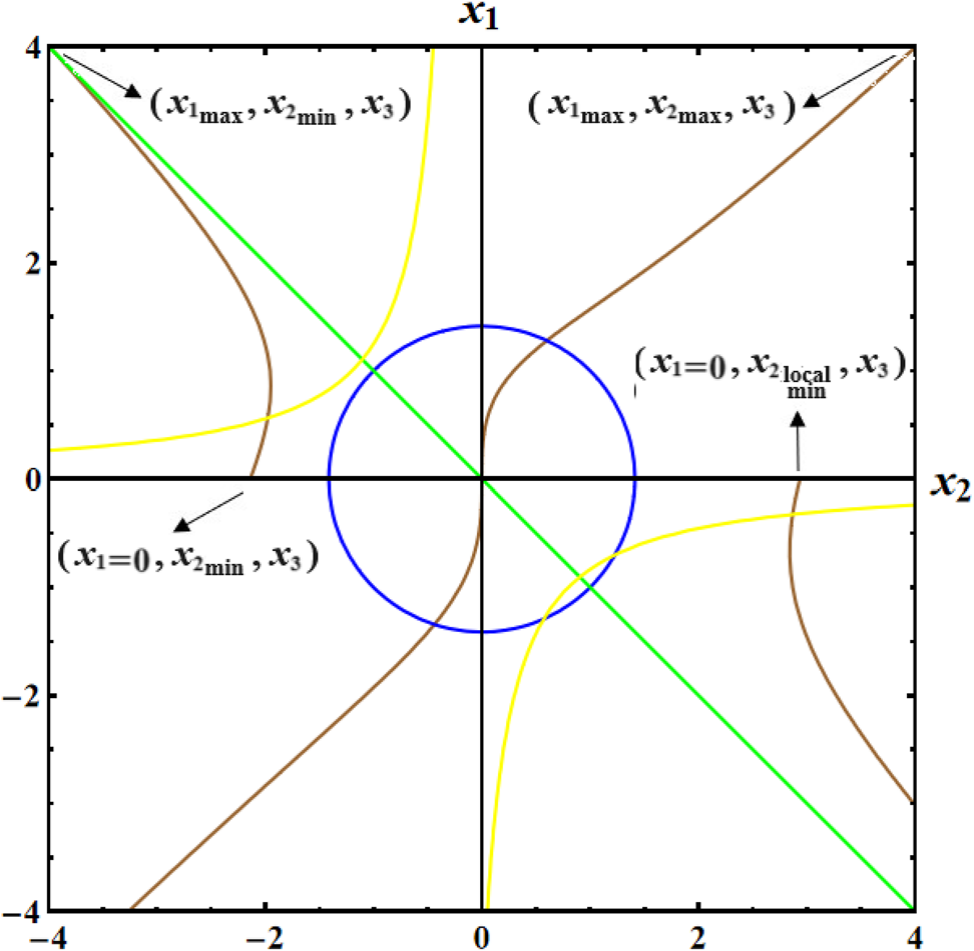
plots of typical steady trajectories for a constant minor torque axis.

The SP, CP, and VC connected to the LS surface converge into a single focal point at $(x_{1} ,x_{2} ,x_{3} ) = (1, - 1,0)$, whereas those connected to the RS surface converge into $(x_{1} ,x_{2} ,x_{3} ) = ( - 1,1,0)$. The statement holds true in the case where $F = 1.41$. So, starting with $F = 1.41$ in Fig. 3, one may create the SP, CP, and VC curves by tracing the polar coordinates $(F,\psi_{1} = {\alpha \mathord{\left/ {\vphantom {\alpha 2}} \right. \kern-0pt} 2})$ covered by the SP, CP, and VC points as $F$ is raised. The motion that started from the separatrix area will stay trapped there and approach steadily upon the EP in the location of the intersection of $(x_{1}^{2} + x_{2}^{2} )^{2} - 4 = 0$ with the unstable branch of the hyperbola $x_{2} (x_{1} + b) = - 1$, which is encapsulated in that area. The projection of the trajectories on the $(x_{1} ,x_{2} )$ plane forms a segment of a circle with a radius of $F = 1.41$ since it is in the stable zone bounded by the RS surface, which is plotted in Fig. 7.

By modifying system (13), one may determine the extreme values that the dimensionless angular velocity component $x_{1}$, $x_{2}$, and $x_{3}$ takes along an enclosed trajectory in each of the two regions of stability with constant torque along the minor axis as follows

34 $$\begin{gathered} \frac{{dx_{1} }}{{dx_{2} }} = \frac{{ - x_{2} }}{{x_{1} + a}} = 0,\,\,\,\,\,\,\,\,\,\,\,\,\,\,\,\,\,\,\,\,\,\,\,\,\,\,\frac{{dx_{2} }}{{dx_{1} }} = \frac{{ - (x_{1} + a)}}{{x_{2} }} = 0, \hfill \\ \frac{{dx_{1} }}{{dx_{3} }} = \frac{{x_{3} }}{{x_{1} + b}} = 0,\,\,\,\,\,\,\,\,\,\,\,\,\,\,\,\,\,\,\,\,\,\,\,\,\,\,\frac{{dx_{3} }}{{dx_{1} }} = \frac{{x_{1} + b}}{{x_{3} }} = 0,\,\, \hfill \\ \frac{{dx_{2} }}{{dx_{3} }} = \frac{{ - x_{3} (x_{1} + a)}}{{x_{2} (x_{1} + b)}} = 0,\,\,\,\,\,\,\,\,\,\,\,\,\,\frac{{dx_{3} }}{{dx_{2} }} = \frac{{ - x_{2} (x_{1} + b)}}{{x_{3} (x_{1} + a)}} = 0. \hfill \\ \end{gathered}$$

It's crucial to mention that the extremal values for $x_{1}$ and $x_{2}$ will only take place when the dimensionless angular velocity path intersects the plane $(x_{1} ,x_{2} )$ or the plane $(x_{1} ,x_{3} )$. In contrast, the extreme values for $x_{3}$ will only take place when this trajectory intersects with the two surfaces $x_{2} (x_{1} + b) = - 1$, which is a direct result of the system (34). By replacing the formulas for the constants of the motion $F$ and $G_{1}$, provided by Eqs. (18) and (30), with the system (34), and calculating the resultant transcendental equations, it is possible to determine the extremes for $x_{1} ,x_{2} ,$ and $x_{3}$.

Considering the following initial data to continue our estimation as the initial values of the scaled angular velocity components $x(0) = (2.5,4,0.1)$, the values of the principal inertia $D( = 2000,1500,1000){\text{kg}}\;{\text{m}}^{2}$, and the value of the first component of the GM $\lambda_{1} ( = 100,300,500){\text{kg}}\;{\text{m}}^{2} \;{\text{s}}^{ - 1}$. Then, the values for both constant of the motion are $F = 4.72$ and $G_{1} = 2.54$.

As a result, Eq. (18) implies

35 $$x_{1} = \pm \sqrt {F^{2} - x_{2}^{2} } ,\,\,\,\,\,x_{2} = \pm \sqrt {F^{2} - x_{1}^{2} } .$$

The substitution of (30) into (35) produces

36 $$2x_{3}^{2} - 2x_{1}^{2} - 4bx_{1} \pm 4\tan^{ - 1} ({{x_{1} } \mathord{\left/ {\vphantom {{x_{1} } {\sqrt {F^{2} - x_{1}^{2} } }}} \right. \kern-0pt} {\sqrt {F^{2} - x_{1}^{2} } }}) = G_{1} \pm F^{2} ,$$

37 $$2x_{3}^{2} + 2x_{2}^{2} \pm 4b\sqrt {F^{2} - x_{2}^{2} } \pm 4\tan^{ - 1} ({{\sqrt {F^{2} - x_{2}^{2} } } \mathord{\left/ {\vphantom {{\sqrt {F^{2} - x_{2}^{2} } } {x_{2} }}} \right. \kern-0pt} {x_{2} }}) = G_{1} \pm F^{2} .$$

The conditions associated with the $x_{3}$ extremes are provided by

38 $$x_{2} (x_{1} + b) = - 1.$$

Equation (38) is substituted into Eq. (18) to get

39 $$x_{1}^{4} + 2bx_{1}^{3} + (b^{2} - F^{2} )x_{1}^{2} - 2bF^{2} x_{1} - b^{2} F^{2} + 1 = 0,$$

40 $$x_{2}^{4} + (b^{2} - F^{2} )x_{2}^{2} - 2bx_{2} + 1 = 0.$$

Then substituting the previous solutions for $x_{1}$ and $x_{2}$ into Eq. (30) to produce the required extreme values of $x_{3}$.

Table 2 provides an analytical statement for the extreme values of several dimensionless angular velocity components $x_{1} ,x_{2} ,$ and $x_{3}$ alongside a solution in CBFT along minor axis which is periodic.

**Table 2:** Presents $x_{j}$ extreme values corresponding to the minor axis as $(\mu_{1} ,\mu_{2} ,\mu_{3} ) = (0,0,1)$.

Item	Analytic statement	Estimated values
General	$F = \sqrt {x_{1}^{2} (0) + x_{2}^{2} (0)} ,$ $G_{1} = 2x_{3}^{2} (0) + x_{2}^{2} (0) - x_{1}^{2} (0) - 4bx_{1} (0) - 4\tan^{ - 1} [\frac{{x_{1} (0)}}{{x_{2} (0)}}].$	$x(0) = (2.5,4,0.1),$ $F = 4.72,\,\,G_{1} = 2.54.$
$x_{{3_{\max } }} ,$ $x_{{3_{\min } }}$	$x_{1} = \frac{{\sqrt {F^{2} - \sqrt {F^{2} - 4} } }}{2},\,\,x_{2} = - \frac{{\sqrt {F^{2} - \sqrt {F^{2} - 4} } }}{2},$ $x_{3} = \pm \sqrt {\frac{{G_{1} - x_{2}^{2} + x_{1}^{2} + 4bx_{1} + 4\tan^{ - 1} ({{x_{1} } \mathord{\left/ {\vphantom {{x_{1} } {x_{2} }}} \right. \kern-0pt} {x_{2} }})}}{2}} .$	$x = (3.64, - 2.99, \pm 1.76).$
$x_{{2_{\max } }}$	$x_{1} = 0,\,\,x_{2} = F,$ $x_{3} = \pm \sqrt {{{(G_{1} + 2F^{2} + 4\pi )} \mathord{\left/ {\vphantom {{(G_{1} + 2F^{2} + 4\pi )} 2}} \right. \kern-0pt} 2}} .$	$x = (0,4.72, \pm 5.56).$
$x_{{1_{\max } }}$	$x_{1} = F,\,\,x_{2} = 0,$ $x_{3} = \pm \sqrt {{{(G_{1} + 2F^{2} + 4bF + 4\pi )} \mathord{\left/ {\vphantom {{(G_{1} + 2F^{2} + 4bF + 4\pi )} 2}} \right. \kern-0pt} 2}} .$	$x = (4.72,0, \pm 5.44).$

## CBFT along the major axis

In this section, an approximate solution for the angular velocities of the RB when it is subjected to CBFT along its major axis is explored. This method is employed when the analytical solution fails to be achieved and will be further discussed later on. If the constant positive torque along the major axis has the form $(\mu_{1} ,\mu_{2} ,\mu_{3} ) = (1,0,0)$, then system (5) can be rewritten as follows

41 $$\begin{aligned} \frac{{dx_{1} }}{d\tau } - x_{2} x_{3} & = 1, \\ \frac{{dx_{2} }}{d\tau } + x_{3} x_{1} + ax_{3} & = 0, \\ \frac{{dx_{3} }}{d\tau } - x_{1} x_{2} - bx_{2} & = 0. \\ \end{aligned}$$

The equations presented in the above system illustrate their EPs, wherein they manifest as a hyperbola with the equation $X_{2} X_{3} = - 1$. Additionally, they are stable at $X_{3} > 1$ and unstable at $\left| {X_{2} } \right| > 1$. Adding a new variable $\alpha_{2}$ through the following equation as

42 $$\alpha_{2} (\tau ) = \int\limits_{0}^{\tau } {x_{1} (\sigma_{2} )\,d\sigma_{2} } ,$$

which also has the form ${{d\alpha_{2} } \mathord{\left/ {\vphantom {{d\alpha_{2} } {d\tau }}} \right. \kern-0pt} {d\tau }} = x_{1} (\tau );\,\,\,\alpha_{2} (0) = 0$. Applying this variable will transform the system (41) into

43 $$\begin{aligned} \frac{{dx_{1} }}{{d\alpha_{1} }} - x_{2} & = 0, \\ \frac{{dx_{2} }}{{d\alpha_{1} }} + x_{1} + a & = 0, \\ \frac{{d^{2} \alpha_{1} }}{{d\tau^{2} }} - x_{1} x_{2} - bx_{2} & = 1. \\ \end{aligned}$$

It is significant to note that the variables of system (43) can't be separated, and then it is impossible to obtain the analytical solution of this system by using the aforementioned method. Therefore, a numerical solution for the system is going to be presented and diagrammed to see the various effects of the GM on the RB motion. All numerical simulations were performed concerning the aforementioned values of the inertia’s main moments $D( = 2000,1500,1000){\text{kg}}{\text{.m}}^{2}$ besides the values the GM $\lambda_{1} ( = 100,300,500)\,{\text{kg}}{\text{.m}}^{2} {\text{.s}}^{ - 1}$.

Currently, the rotational motion of the RB, with the assumption that the torque along the major axis remains constant, is going to be examined. In this particular scenario, Figs. 8, 9, 10, 11, 12, 13, 14, 15 are drawn to depict the equilibrium conditions on the specific regions mentioned in Table 1.

**Figure 8:**
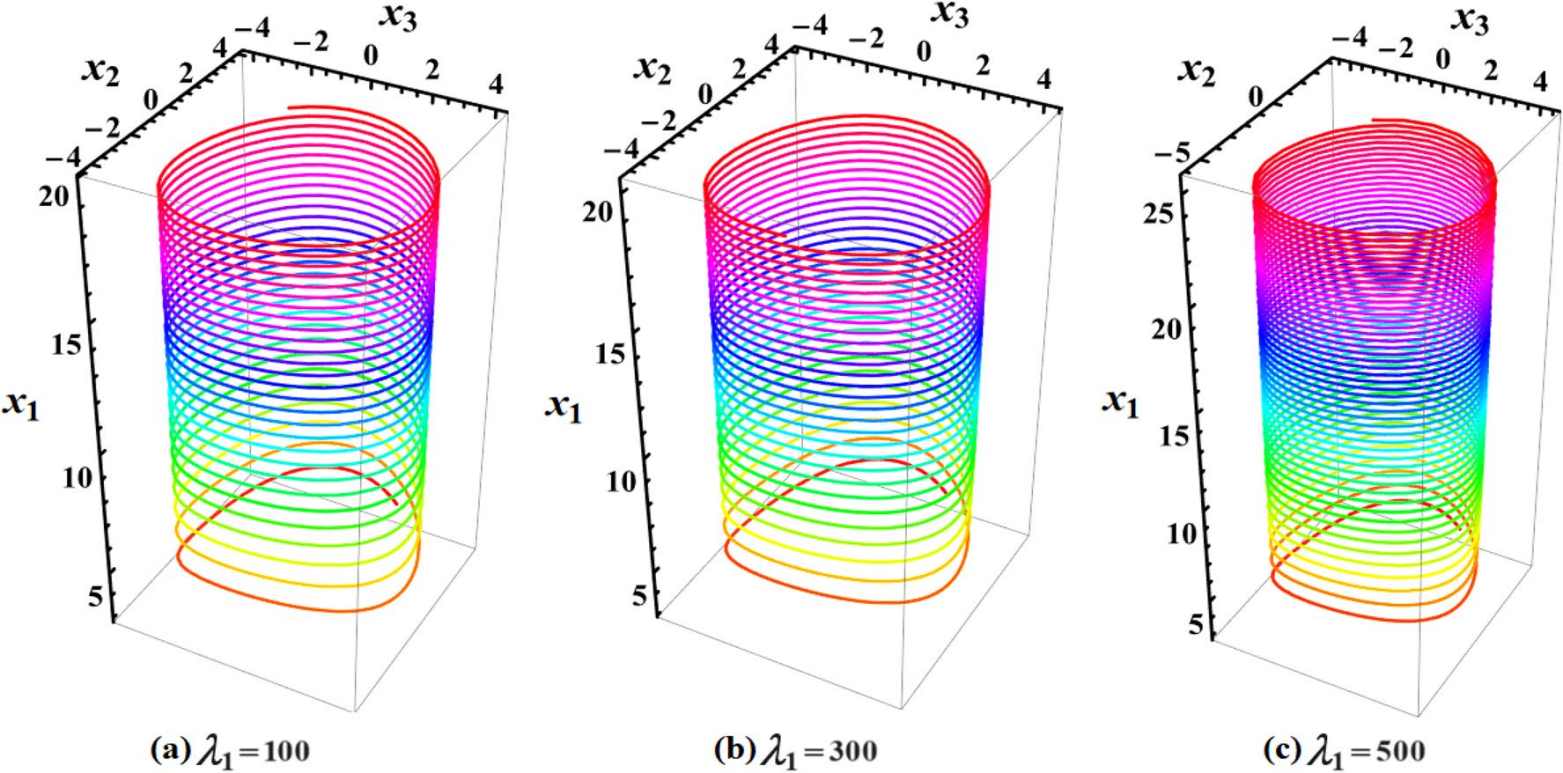
Presents a 3D plot for $x_{1} ,x_{2} ,$ and $x_{3}$ at a constant major axis torque when $\lambda_{1} ( = 100,300,500)\;{\text{kg}}\;{\text{m}}^{2} \;{\text{s}}^{ - 1}$.

**Figure 9:**
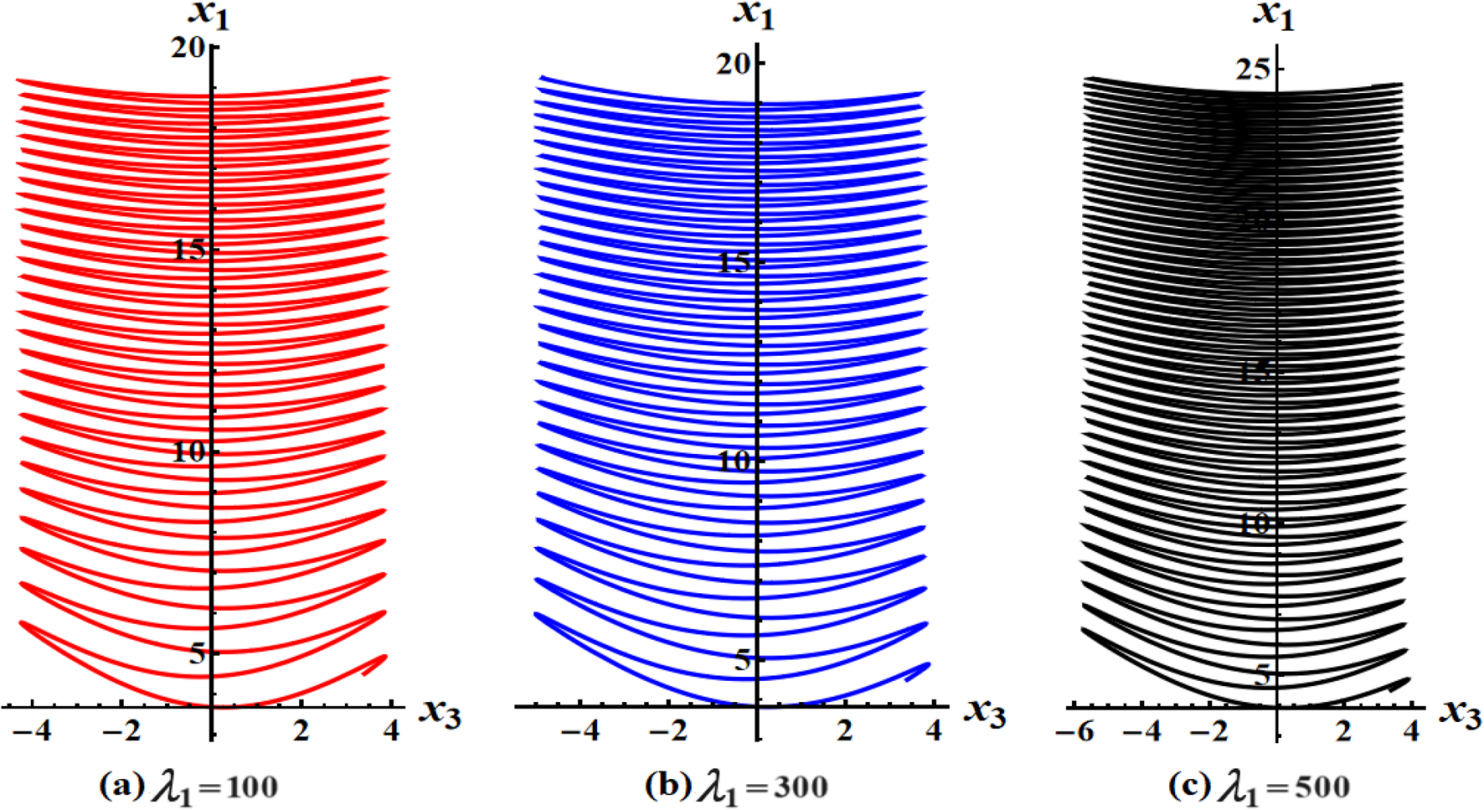
Shows 2D plots for the represented angular velocities $x_{1}$ and $x_{3}$ in Fig. 8.

**Figure 10:**
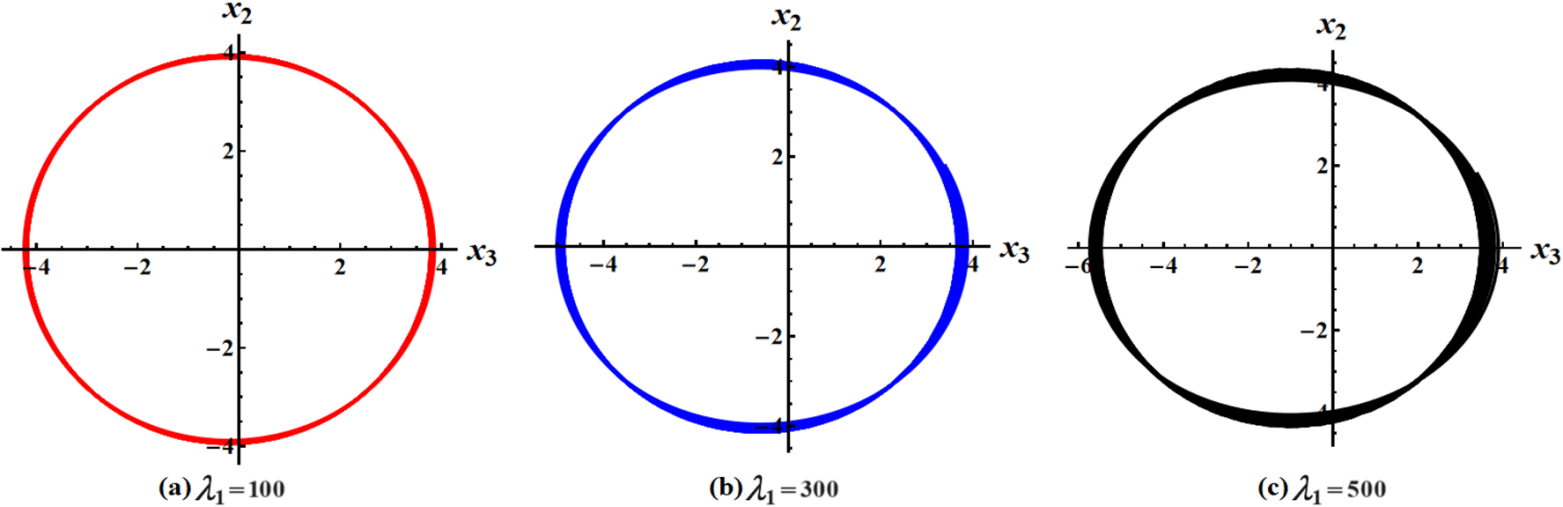
Shows 2D plots for the given angular velocities $x_{2}$ and $x_{3}$ in Fig. 8.

**Figure 11:**
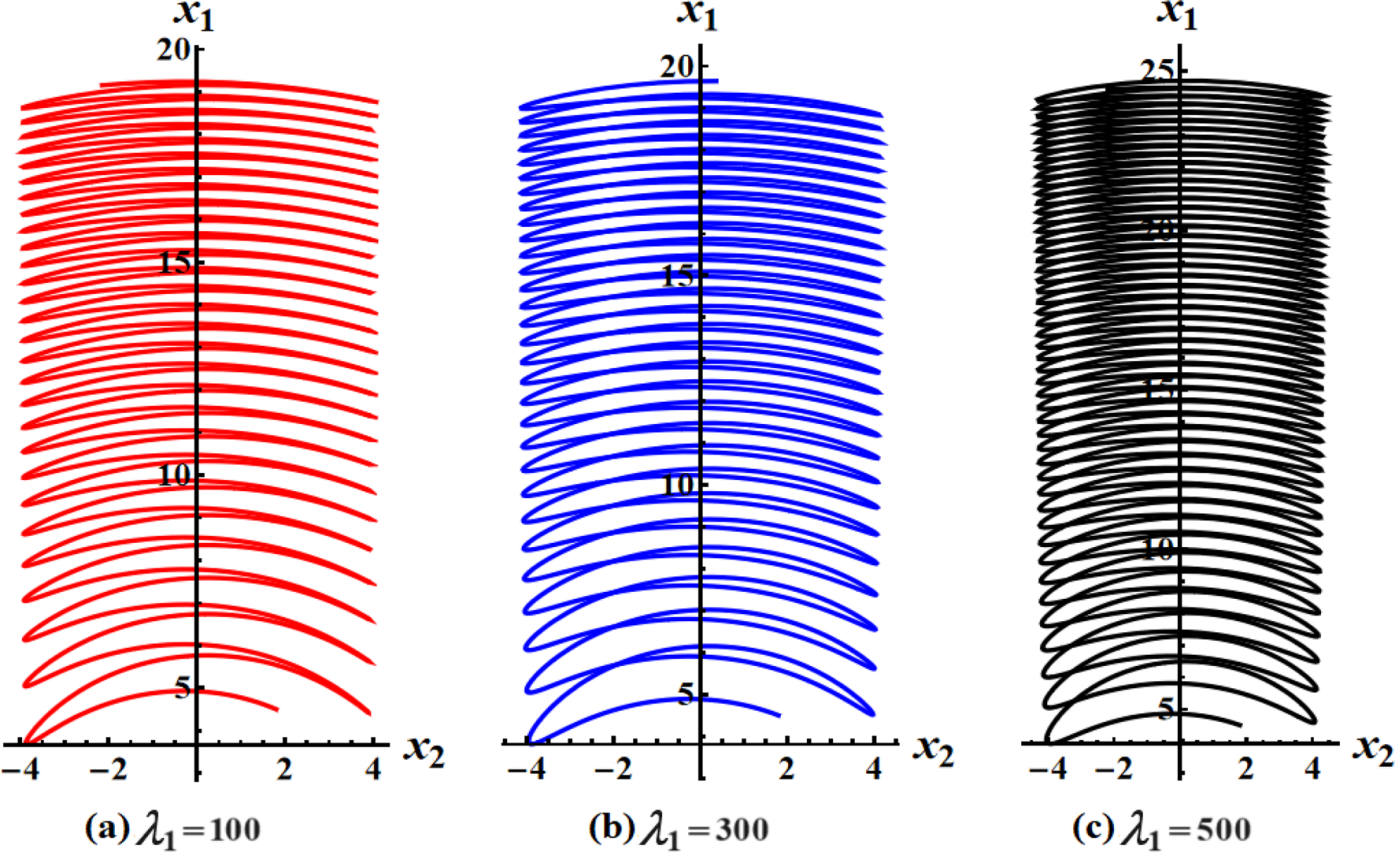
Shows 2D plots for the graphed angular velocities $x_{2}$ and $x_{3}$ in Fig. 8.

**Figure 12:**
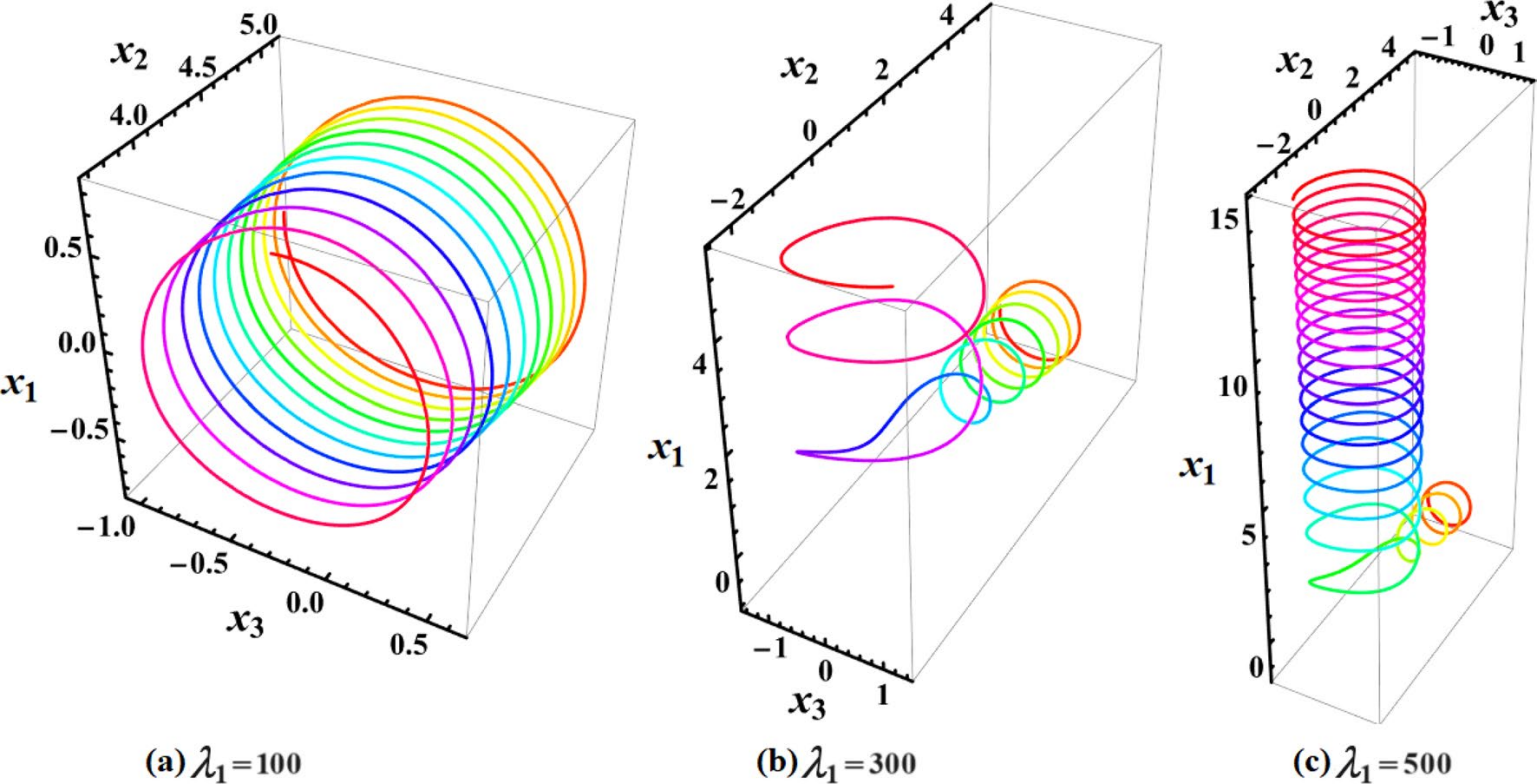
Expresses 3D plots of $x_{j} \,(j = 1,2,3)$ at a constant major axis torque when $\lambda_{1} ( = 100,300,500)\;{\text{kg}}\;{\text{m}}^{2} \;{\text{s}}^{ - 1}$.

**Figure 13:**
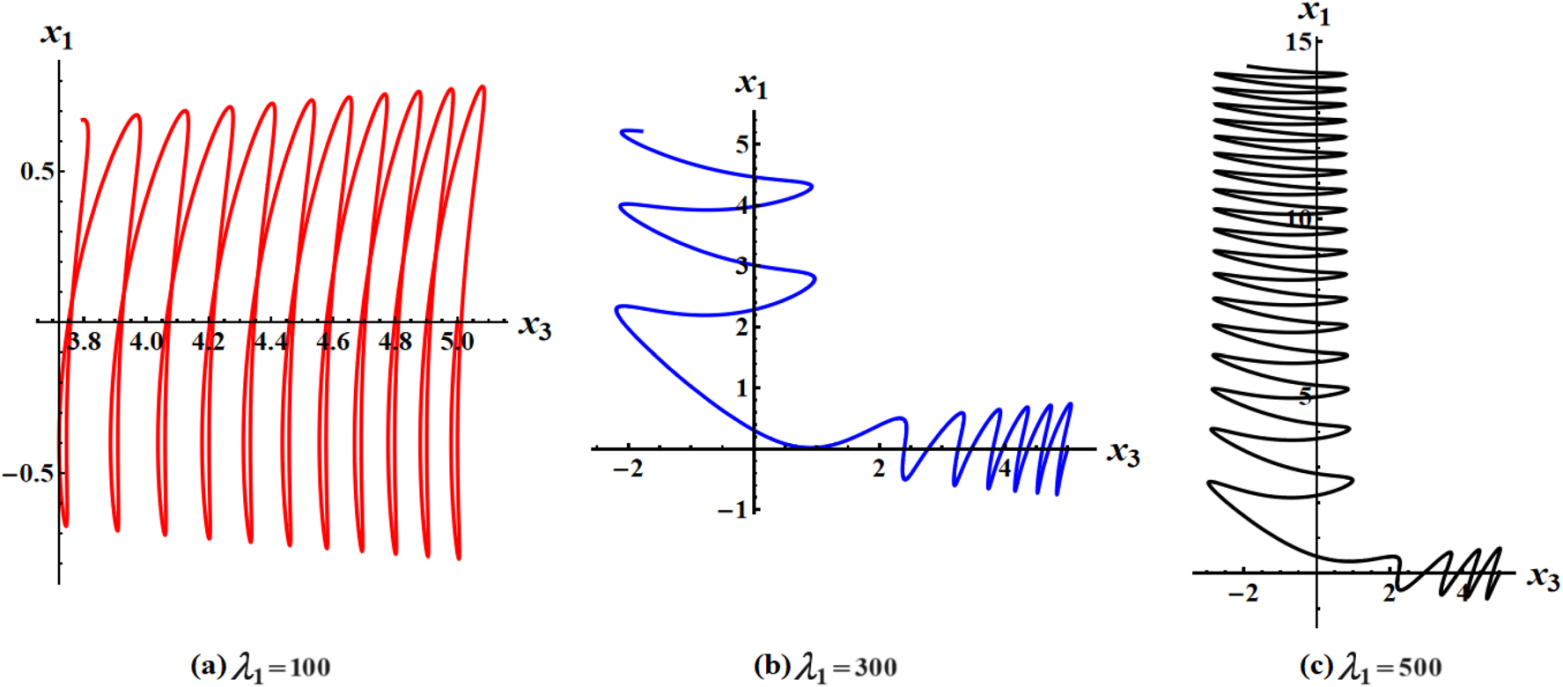
Plots 2D graphs for the drawn angular velocities $x_{1}$ and $x_{3}$ in Fig. 12.

**Figure 14:**
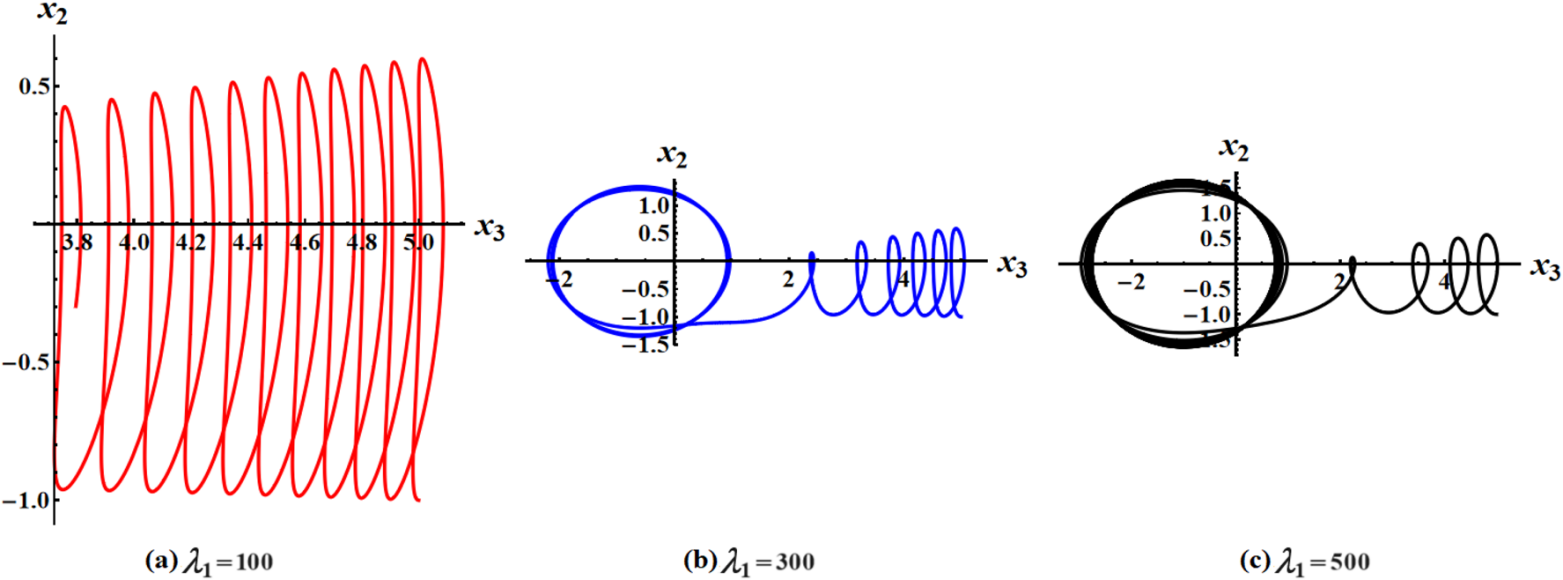
Plots 2D graphs for the drawn angular velocities $x_{2}$ and $x_{3}$ in Fig. 12.

**Figure 15:**
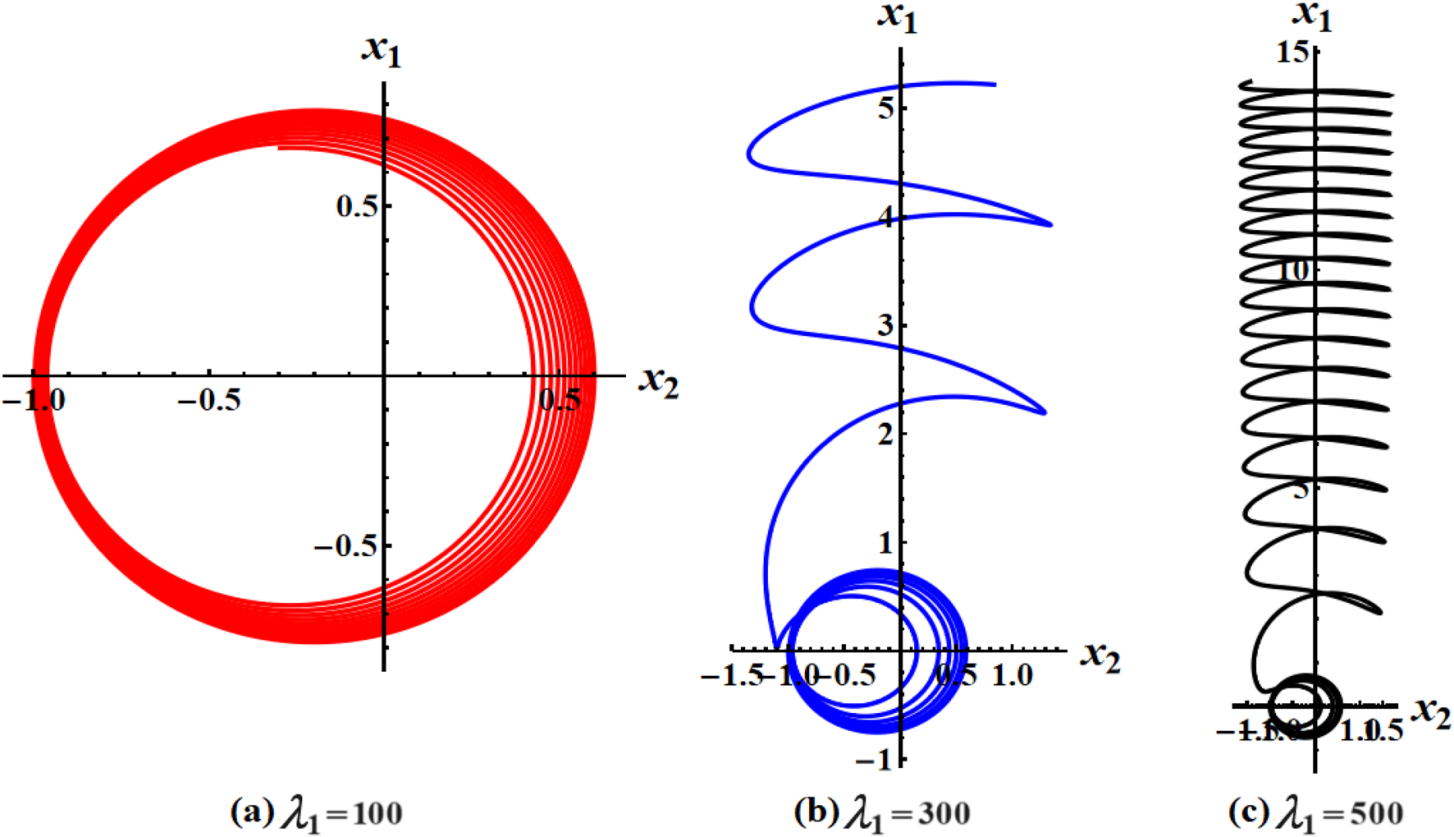
Plots 2D graphs for the drawn angular velocities $x_{1}$ and $x_{2}$ in Fig. 12.

Figure 8 describes the 3D trajectory of the angular velocities $x_{1} ,x_{2} ,$ and $x_{3}$ of the RB when the GM has the values $\lambda_{1} ( = 100,300,500)\,{\text{kg}}\,{\text{m}}^{2}\,{\text{s}}^{ - 1}$. On the other hand, the projections in the planes $x_{1} x_{3} ,\,\,x_{2} x_{3} ,$ and $x_{1} x_{2}$ are presented in Figs. 9, 10, and 11, respectively, at the same values as Fig. 8. These figures are generated with the initial condition $x(0) = (4.5,1.8,3.4)$ at the upper unstable region of the space $(x_{1} x_{2} x_{3} )$ during the interval $\tau \in [0,15]$ ${\text{s}}$.

Note that in all 3D graphical simulations, the motion's trajectory begins with rainbow colors and then transitions to a unique color during its path interval.

In Fig. 8, as the GM value equals $100\;{\text{kg}}\;{\text{m}}^{2} \;{\text{s}}^{ - 1}$, the body begins its spin in the positive area of $x_{1}$ and remains spinning between the intervals $x_{2} \in [ - 4,4]$ and $x_{3} \in [ - 4.1,4]$ for its oscillation, where it eventually converges on a spin-up maneuver. As the GM value approaches to the value $300\;{\text{kg}}\;{\text{m}}^{2} \;{\text{s}}^{ - 1}$, it’s released that the body follows the same path as before, but it is noticed that an increase in the amplitude of the spin maneuver in the positive region of $x_{1}$ towards the negative regions of $x_{1} ,x_{2} ,$ and $x_{3}$, as seen in the 2D simulations of Fig. 9, 10, 11. The increasing of the frequency of the body oscillation increases the oscillation interval as $x_{2} \in [ - 4.2,4]$ and $x_{3} \in [ - 5,4]$. At $\lambda_{1} = 500{\text{kg}}{\text{.m}}^{2} {\text{.s}}^{ - 1}$, the body spins increasingly to maneuver around $x_{1}$. The amplitude of the maneuver about $x_{2}$ and $x_{3}$ is also increases as $x_{2} \in [ - 4.5,4]$ and $x_{3} \in [ - 6,4]$, and then the closed paths of the trajectories keep it spinning manner with a slight deformation for the one path trajectory before the increasing of the GM value, as shown in Fig. 10. The importance of these outcomes lies in their broad utilization across various domains. For instance, they can be employed to stabilize the movement of spacecraft in their orbits. This is achieved by adjusting the GM value, which impacts the component of the body's angular velocity and ensures it remains within the designated orbit.

Figures 12, 13, 14, 15 are calculated at the stable region of the space $(x_{1} x_{2} x_{3} )$ during the interval $\tau \in [0,15]{\text{s}}$ when the initial condition $x(0) = (0, - 1,5)$ is considered. The outcomes of this simulation are graphed at distinct values of the first component of the GM $\lambda_{1}$. At $\lambda_{1} = 100\;{\text{kg}}\;{\text{m}}^{2} \;{\text{s}}^{ - 1}$, the trajectory initiates a customary rotational acceleration maneuver inside positive region of angular velocity $x_{3}$ with a slight decrease in its amplitude towards the negative region and the body has a stable motion, as explored in Fig. 12a. As the GM increases to become $300\;{\text{kg}}\;{\text{m}}^{2} \;{\text{s}}^{ - 1}$, the body starts its spinning motion about the positive stable region of $x_{3}$, and then moves towards the SS with a higher oscillation amplitude and lower frequency. Therefore, it crosses into the positive unstable region of $x_{1}$, where it converges to a pure spin-up maneuver, as shown in Fig. 12b. At $\lambda_{1} = 500\;{\text{kg}}\;{\text{m}}^{2} \;{\text{s}}^{ - 1}$, an increase in the amplitude of the spinning about the negative region of $x_{1}$ is noticed, and the trajectory follows the same path as $\lambda_{1} = 300\;{\text{kg}}\;{\text{m}}^{2} \;{\text{s}}^{ - 1}$ with the same motion's behavior, as observed in Fig. 12c. This description can be easily noticed in the 2D representation of the body's angular velocities, as seen in Figs. 13, 14, 15.

## CBFT along the middle axis

In this section, the analytical method employed to calculate the angular velocities of the RB while it experiences CBFT along its middle axis, is explored. In the scenario, we consider $(\mu_{1} ,\mu_{2} ,\mu_{3} ) = (0,1,0)$ for this torque along the middle axis, and then equations of system (5) provide the following form

44 $$\begin{gathered} \frac{{dx_{1} }}{d\tau } - x_{2} x_{3} = 0, \hfill \\ \frac{{dx_{2} }}{d\tau } + x_{3} x_{1} + ax_{3} = 1, \hfill \\ \frac{{dx_{3} }}{d\tau } - x_{1} x_{2} - bx_{2} = 0. \hfill \\ \end{gathered}$$

In this scenario, the points of equilibrium in the system form a hyperbola resembling the form $(X_{1} + a)X_{3} = 1$. As mentioned in the preceding three sections, we consider a new variable $\alpha_{3}$ such that

45 $$\alpha_{3} (\tau ) = \int\limits_{0}^{\tau } {x_{2} (\sigma_{3} )\,d\sigma_{3} } ,$$

In other words, one can write this formula as ${{d\alpha_{3} } \mathord{\left/ {\vphantom {{d\alpha_{3} } {d\tau }}} \right. \kern-0pt} {d\tau }} = x_{2} (\tau );\,\,\,\alpha_{3} (0) = 0$. Applying this transformation to the system (44), yields

46 $$\begin{gathered} \frac{{dx_{1} }}{{d\alpha_{3} }} - x_{3} = 0, \hfill \\ \frac{{d^{2} \alpha_{3} }}{{d\tau^{2} }} + x_{1} x_{3} + ax_{3} = 1, \hfill \\ \frac{{dx_{3} }}{{d\alpha_{3} }} - x_{1} - b = 0. \hfill \\ \end{gathered}$$

The solutions to the first and third equations of system (46) have the forms

47 $$\begin{gathered} x_{1} = b + x_{3} (0)\sinh \alpha_{3} + x_{1} (0)\cosh \alpha_{3} , \hfill \\ x_{3} = x_{3} (0)\cosh \alpha_{3} + x_{1} (0)\sinh \alpha_{3} , \hfill \\ \end{gathered}$$

where they satisfy the next equation

48 $$x_{3}^{2} - x_{1}^{2} = x_{3}^{2} (0) - x_{1}^{2} (0) - b\{ b + 2[x_{3} (0)\sinh \alpha_{3} + x_{1} (0)\cosh \alpha_{3} ]\} .$$

Substituting Eq. (47) into the second equation of system (46) to get

49 $$\begin{gathered} \frac{{d^{2} \alpha_{3} }}{{d\tau^{2} }} = 1 - (a + b)[x_{1} (0)\sinh \alpha_{3} + x_{3} (0)\cosh \alpha_{3} ] - \frac{{x_{1}^{2} (0) + x_{3}^{2} (0)}}{2}\sinh 2\alpha_{3} \hfill \\ \,\,\,\,\,\,\,\,\,\,\,\,\, - x_{1} (0)x_{3} (0)\cosh 2\alpha_{3} . \hfill \\ \end{gathered}$$

Considering

50 $$L = \frac{1}{2}[x_{1}^{2} (0) + x_{3}^{2} (0)],\,\,\,\,\,M = x_{1} (0)x_{3} (0).$$

Then, Eq. (49) will be

51 $$\frac{{d^{2} \alpha_{3} }}{{d\tau^{2} }} = 1 - L\sinh 2\alpha_{3} - M\cosh 2\alpha_{3} - (a + b)[x_{1} (0)\sinh \alpha_{3} + x_{3} (0)\cosh \alpha_{3} ].$$

**In the trajectories' case with** $\left| {x_{1} (0)} \right| \ne \left| {x_{3} (0)} \right|$**:**

When $L > M$, Eq. (51) takes the form

52 $$\frac{{d^{2} \alpha_{3} }}{{d\tau^{2} }} = 1 - \sqrt {L^{2} - M^{2} } \sinh (2\alpha_{3} + \psi_{2} ) - (a + b)\sqrt {2N} \sinh (\alpha_{3} + \psi_{2} ).$$

where

53 $$\psi_{2} = \tanh^{ - 1} ({M \mathord{\left/ {\vphantom {M L}} \right. \kern-0pt} L}),\,\,\,\,\,N = \frac{1}{2}[x_{1}^{2} (0) - x_{3}^{2} (0)].$$

Considering the following variables

54 $$\begin{gathered} \gamma = 2\alpha_{3} + \psi_{2} , \hfill \\ L^{2} = M^{2} + N^{2} . \hfill \\ \end{gathered}$$

Therefore, one can rewrite Eq. (52) as follows

55 $$\frac{{d^{2} \gamma }}{{d\tau^{2} }} = 2\{ 1 - N\sinh \gamma - (a + b)\sqrt {2N} \sinh (\frac{{\gamma + \psi_{2} }}{2})\} ,$$

where $N$ is the first integral of the motion. Since Eq. (55) represents a conservative system, then at an EP $\gamma = \gamma^{*}$ and $\psi_{2} = \gamma^{*}$ as $\gamma^{*} = \alpha_{3} (\tau ) = \alpha_{3} (0) = \psi_{2}$, one can obtain

56 $$\gamma^{ * } = \sinh^{ - 1} [\frac{1}{{N + (a + b)\sqrt {2N} }}].$$

Implying that $\psi_{2} = \gamma^{*}$, led to

57 $$\sinh \psi_{2} = \frac{M}{L},$$

Hence,

58 $$M = \frac{N}{{N + (a + b)\sqrt {2N} }},$$

which implies that

59 $$M = x_{1} (0)x_{3} (0) = X_{1} X_{3} = \frac{N}{{N + (a + b)\sqrt {2N} }},$$

Recalling the equation

60 $$X_{3} (X_{1} + a) = 1,$$

which holds at EPs. Therefore, the substitution of $X_{3}$ from (59) into the hyperbola’s Eq. (60) yields

61 $$X_{1} = \frac{a}{(a + b)}\sqrt{\frac{N}{2}} .$$

Now, Eq. (60) implies that

62 $$X_{3} = \frac{a + b}{{a(\sqrt {{N \mathord{\left/ {\vphantom {N 2}} \right. \kern-0pt} 2}} + a + b)}}.$$

Then, the first EP for the system is $(\frac{a}{a + b}\sqrt{\frac{N}{2}} ,\,\,0,\,\,\frac{a + b}{{a(\sqrt {{N \mathord{\left/ {\vphantom {N 2}} \right. \kern-0pt} 2}} + a + b)}})$.

The system (44) has a constant amount of energy due to the fact that Eq. (55) can be perceived as a force field^3^ that conservatively acts on the right-hand side. In order to determine this constant, it is necessary to rewrite Eq. (55) as follows

63 $$\frac{d\gamma }{{d\tau }} = y.$$

Making use of Eq. (55) to yield

64 $$\frac{dy}{{d\tau }} = 2\left[ {1 - N\sinh \gamma - (a + b)\sqrt {2N} \sinh \left( {\frac{{\gamma + \psi_{2} }}{2}} \right)} \right].$$

Dividing the previous two equations to get

65 $$\gamma \frac{dy}{{d\gamma }} = 2\left[ {1 - N\sinh \gamma - (a + b)\sqrt {2N} \sinh \left( {\frac{{\gamma + \psi_{2} }}{2}} \right)} \right].$$

Integration of this equation, yields

66 $$\frac{{y^{2} }}{2} + 2\left[ {N\cosh \gamma + 2(a + b)\sqrt {2N} \cosh \left( {\frac{{\gamma + \psi_{2} }}{2}} \right) - \gamma } \right] = G_{2} ,$$

where the second integral of motion is donated by $G_{2}$ and is defined as the system’s dimensionless energy constant.

The dimensionless variable representing potential energy (distinct from actual potential energy) within the system may be defined according to

67 $$V = 2\left[ {N\cosh \gamma + 2(a + b)\sqrt {2N} \cosh \left( {\frac{{\gamma + \psi_{2} }}{2}} \right) - \gamma } \right].$$

Hence,

68 $$\frac{{d^{2} V}}{{d\gamma^{2} }} = 2N\cosh \gamma + (a + b)\sqrt {2N} \cosh \left( {\frac{{\gamma + \psi_{2} }}{2}} \right) > 0.$$

It is easy to deduce that the EP $\gamma^{*}$ is stable. This indicates that every starting condition where $\left| {x_{1} (0)} \right| \ne \left| {x_{3} (0)} \right|$ produces a path that is an enclosed periodic. It has a shape that is elliptical around a distinct EP that is stable according to Lyapunov's criterion.

As every point on the pathways is recognized as a set of initial conditions for it, at $\gamma (0) = \psi_{2}$, Eqs. (55) and (68) yields

69 $$2N\cosh \gamma + 4(a + b)\sqrt {2N} \cosh (\frac{{\gamma + \psi_{2} }}{2}) = 2L[1 + 2(a + b)\sqrt {{2 \mathord{\left/ {\vphantom {2 N}} \right. \kern-0pt} N}} ].$$

Also, as $y = 2x_{2}$, Eq. (66) becomes

70 $$2x_{2}^{2} + (x_{1}^{2} + x_{3}^{2} )[1 + 2(a + b)\sqrt {{2 \mathord{\left/ {\vphantom {2 N}} \right. \kern-0pt} N}} ] - 2\tanh^{ - 1} \frac{{2x_{1}^{2} x_{3}^{2} }}{{x_{1}^{2} + x_{3}^{2} }} = G_{2} .$$

Substituting the expression (53) about $N$ to get

71 $$2x_{2}^{2} + (x_{1}^{2} + x_{3}^{2} )[1 + 2(a + b)(x_{1}^{2} - x_{3}^{2} )^{{{{ - 1} \mathord{\left/ {\vphantom {{ - 1} 2}} \right. \kern-0pt} 2}}} ] - 2\tanh^{ - 1} \frac{{2x_{1}^{2} x_{3}^{2} }}{{x_{1}^{2} + x_{3}^{2} }} = G_{2} .$$

The enclosed pathway's 3D simulation, maintaining a constant energy $G_{2}$ is given by Eq. (71). It is calculated for the scenario that involves CBFT along central axis in addition to a starting condition $\left| {x_{1} (0)} \right| \ne \left| {x_{3} (0)} \right|$.

**Figure 16:**
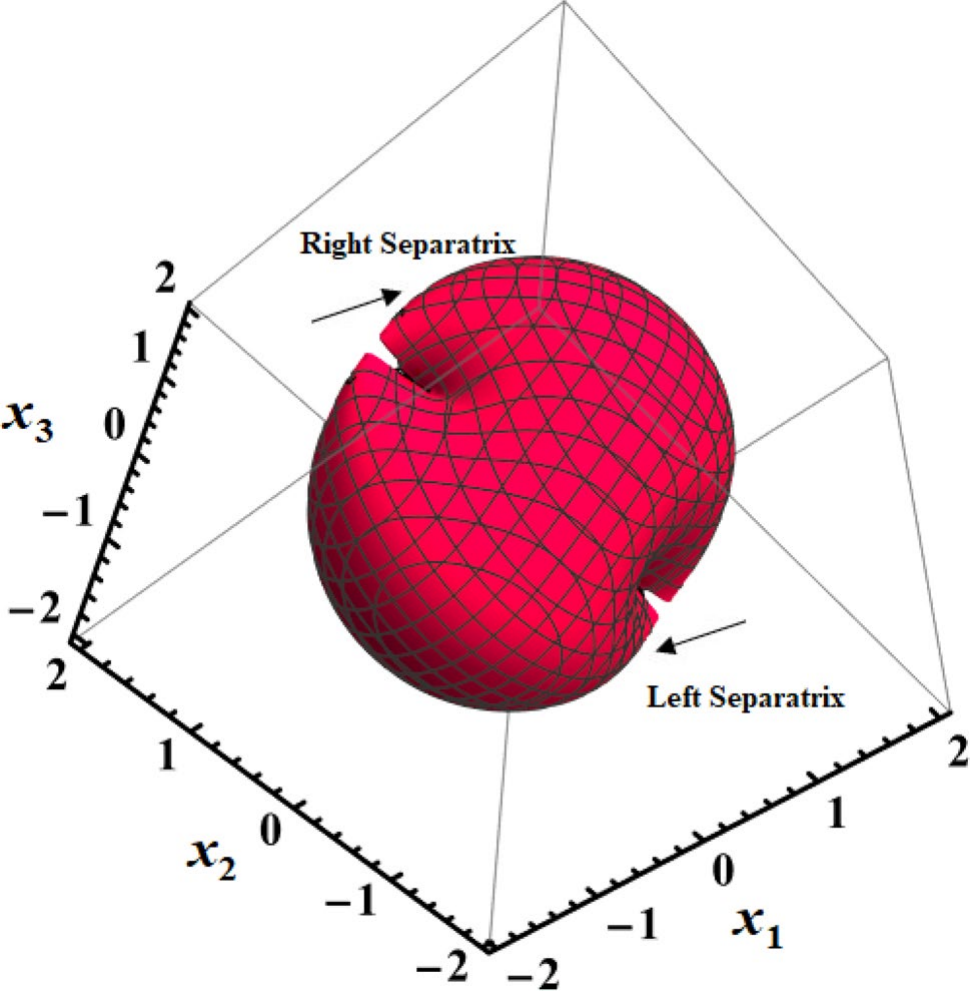
Presents a 3D simulation for SS for a constant middle torque axis.

Figure 16 provides a visual representation of the 3D simulation depicting SS of Eq. (71). These SSs divide the space $(x_{1} x_{2} x_{3} )$ into three infinite regions: two stable regions and an unstable one. Any motion originating from one of these regions will continue to be associated with that region. If the initial conditions for motion lie within either of the stable regions, the trajectory will be closed and restricted, revolving around its corresponding center. On the other hand, if the initial conditions fall within the unstable region, a spin-up maneuver will occur.

**Figure 17:**
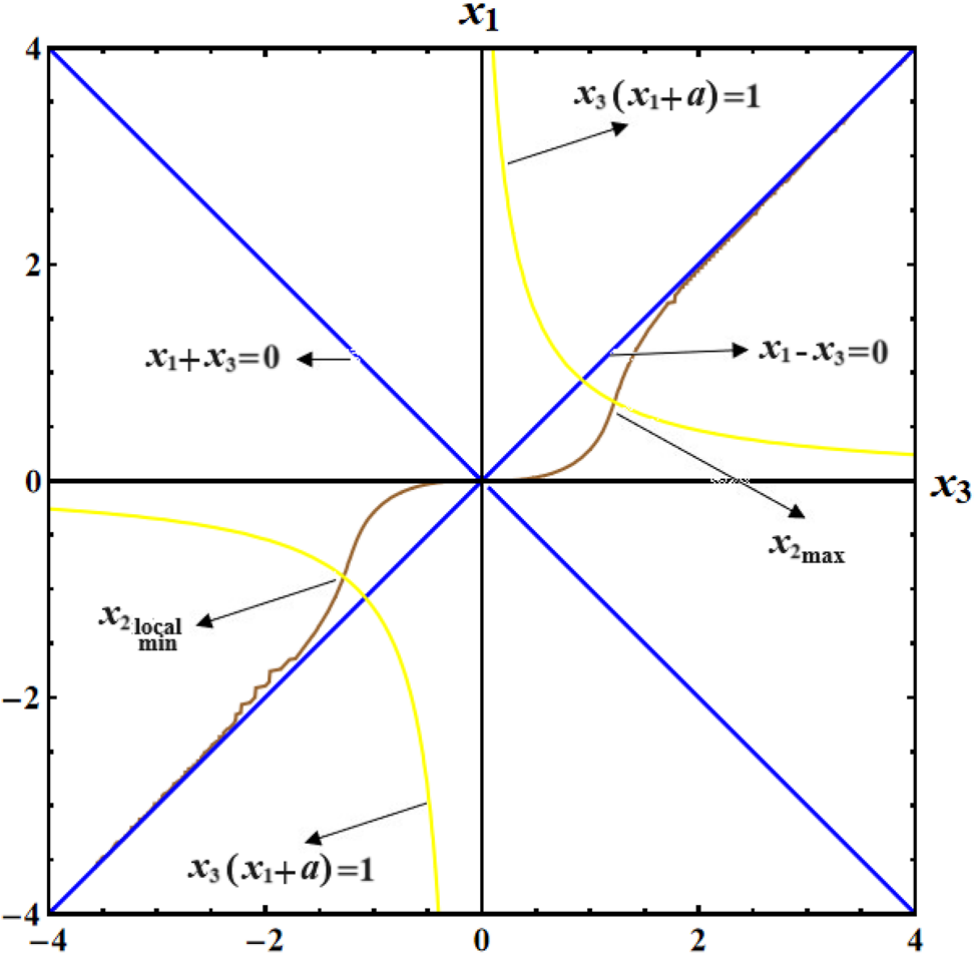
Shows projection of SS in the plane $(x_{1} x_{3} )$ for a constant middle torque axis.

Figure 17 shows the drawing at initial condition $x(0) = (1.7,1.1, - 0.2)$ for the following surfaces:

1. the surface $x_{1}^{2} - x_{3}^{2} = 0$ where none of the EPs are located,
2. the two branches of the hyperbola $x_{3} (x_{1} + a) = 1$, in which all of the system's EPs are located,
3. the line $x_{1} - x_{3} = 0$ that separates the two branches of the hyperbola $x_{3} (x_{1} + a) = 1$ into regions with stable and unstable regions,
4. the surface $$(x_{1}^{2} + x_{3}^{2} )[1 + 2(a + b)(x_{1}^{2} - x_{3}^{2} )^{{{{ - 1} \mathord{\left/ {\vphantom {{ - 1} 2}} \right. \kern-0pt} 2}}} ] - 2\tanh^{ - 1} [{{2x_{1}^{2} x_{3}^{2} } \mathord{\left/ {\vphantom {{2x_{1}^{2} x_{3}^{2} } {(x_{1}^{2} + x_{3}^{2} )}}} \right. \kern-0pt} {(x_{1}^{2} + x_{3}^{2} )}}] = 3.93$$.

Similar to the examination of the situation of CBFTs impacting in the minor axis, $x_{2}$ and $x_{3}$ attain extreme values within an enclosed trajectory fulfilling $\left| {x_{1} (0)} \right| \ne \left| {x_{3} (0)} \right|$ may be derived for the situation of CBFTs impacting in the central axis. Table 3 summarizes outcomes for such analysis for Figs. 16, 17.

**Table 3:** Shows $x_{j}$ extreme values that correspond to the middle axis $(\mu_{1} ,\mu_{2} ,\mu_{3} ) = (0,1,0)$.

Item	Analytic statement	Estimated Values
General	$N = {{\left| {x_{1}^{2} (0) - x_{3}^{2} (0)} \right|} \mathord{\left/ {\vphantom {{\left| {x_{1}^{2} (0) - x_{3}^{2} (0)} \right|} 2}} \right. \kern-0pt} 2},$ $G_{2} = 2x_{2}^{2} (0) + [x_{1}^{2} (0) + x_{3}^{2} (0)][1 + 2(a + b)\sqrt {{2 \mathord{\left/ {\vphantom {2 N}} \right. \kern-0pt} N}} ] - 2\tanh^{ - 1} \frac{{2x_{1}^{2} (0)x_{3}^{2} (0)}}{{x_{1}^{2} (0) + x_{3}^{2} (0)}}.$	$x(0) = (1.7,1.1, - 0.2),$ $N = 1.43,\,\,G_{2} = 8.24.$
$x_{{2_{\max } }} ,$ $x_{{2_{\min } }}$	$x_{1} = \sqrt {\sqrt {N^{2} + 1} + N} ,\,\,\,x_{3} = \sqrt {\sqrt {N^{2} + 1} - N} ,$ $x_{3} = \pm \sqrt {\frac{{G_{1} - x_{2}^{2} + x_{1}^{2} + 4bx_{1} + 4\tan^{ - 1} ({{x_{1} } \mathord{\left/ {\vphantom {{x_{1} } {x_{2} }}} \right. \kern-0pt} {x_{2} }})}}{2}} .$	$x = (1.78, \pm 1.21,0.56).$
$x_{{1_{\max } }} ,$ $x_{{3_{\max } }}$	$x_{2} = 0,$ $x_{1} = \sqrt {x_{3}^{2} + 2N} .$	$x = (1.77,0,0.52).$
$x_{{1_{\min } }}$	$x_{1} = \sqrt {2N} ,\,\,x_{3} = 0,$ $x_{2} = \pm \sqrt {{{\{ G_{2} - \sqrt {2N} [1 + 2(a + b)\sqrt {{2 \mathord{\left/ {\vphantom {2 N}} \right. \kern-0pt} N}} ]\} } \mathord{\left/ {\vphantom {{\{ G_{2} - \sqrt {2N} [1 + 2(a + b)\sqrt {{2 \mathord{\left/ {\vphantom {2 N}} \right. \kern-0pt} N}} ]\} } 2}} \right. \kern-0pt} 2}} .$	$x = (4.72,0, \pm 5.44).$

### Stable separatrix

In the previous case where $x_{1} (0) = x_{3} (0)$, generate a maneuver characterized by spin in an upward direction around the central axis, as demonstrated by system (44). As $x_{1} (0) = x_{3} (0) \ne 0$, equations in (47) yield

72 $$x_{1} = b + x_{1} (0)e^{{\alpha_{3} }} ,\,\,\,\,\,\,\,\,\,\,\,\,\,x_{3} = x_{3} (0)e^{{\alpha_{3} }} .$$

Moreover, equations in (50) give

73 $$L = \frac{1}{2}[x_{1}^{2} (0) + x_{3}^{2} (0)] = x_{1}^{2} (0),\,\,\,\,\,\,\,\,\,\,\,\,\,\,\,M = x_{1} (0)x_{3} (0) = x_{1}^{2} (0).$$

Therefore, $x_{1} = b + x_{3}$ and $L = M$ accordingly $N = \sqrt {L^{2} - M^{2} } = 0$ where $N$ denotes the first integral of motion. As a result, Eq. (51) implies

74 $$\frac{{d^{2} \alpha_{3} }}{{d\tau^{2} }} = 1 - Le^{{2\alpha_{3} }} - (a + b)x_{1} (0)e^{{\alpha_{3} }} .$$

At the equilibrium $\alpha_{3} = 0$, we have

75 $$\begin{gathered} x_{1} = b + x_{1} (0)e^{{\alpha_{3}^{*} }} = b + x_{1} (0)\,\,\,\,\,\, \Rightarrow \,\,\,\,\,\alpha_{3}^{ * } = 0, \hfill \\ x_{3}^{ * } = x_{3} (0)e^{{\alpha_{3}^{*} }} = x_{3} (0)\,\,\,\,\,\,\,\,\,\,\,\,\,\,\,\,\,\,\, \Rightarrow \,\,\,\,\,\alpha_{3}^{ * } = 0. \hfill \\ \end{gathered}$$

From Eq. (60), one writes

76 $$X_{1} = \frac{{ - a \pm \sqrt {a^{2} + 4} }}{2} = X_{3} ,$$

to give an estimation for the EPs. Letting

77 $$\frac{{d\alpha_{3} }}{d\tau } = x_{2} ,$$

then substituting Eq. (77) into (74) to yield

78 $$x_{2} \frac{{dx_{2} }}{{d\alpha_{3} }} = 1 - x_{1}^{2} (0)e^{{2\alpha_{3} }} - (a + b)x_{1} (0)e^{{\alpha_{3} }} .$$

To achieve the desired outcome, one must first separate the variables and subsequently integrate to obtain the following result

79 $$\frac{{x_{1}^{2} }}{2} + \frac{{x_{2}^{2} }}{2} + (a + b)x_{1} - \ln x_{1} = G_{3} .$$

The system’s dimensionless energy constant is denoted by $G_{3}$, which corresponds to the system's second integral of motion.

As performed in the case of $x_{1} (0) \ne x_{3} (0)$, stability analysis shows that every combination of initial conditions concerning with $x_{1} (0) = x_{3} (0) > 0$ leads to a confined oval-shaped closed periodic solution within the plane $x_{1} (0) = x_{3} (0)$ and encircles the EP $(\frac{{ - a + \sqrt {a^{2} + 4} }}{2},0,\frac{{ - a + \sqrt {a^{2} + 4} }}{2})$ contained within this plane, while every set of initial conditions that satisfy $x_{1} (0) = x_{3} (0) < 0$ leads to a confined oval-shaped closed periodic solution within the plane $x_{1} (0) = x_{3} (0)$ and encircles the EP $(\frac{{ - a - \sqrt {a^{2} + 4} }}{2},0,\frac{{ - a - \sqrt {a^{2} + 4} }}{2})$ contained within this plane. Therefore, it can be inferred that, similar to the scenario of $x_{1} (0) \ne x_{3} (0)$, the two extremes of $x_{1} (0)$ corresponding to $x_{3} (0)$ are positioned at the point where the angular velocity trajectory intersects the plane $x_{2} (0) = 0$. Similarly, the two extremes for $x_{2} (0)$ are situated at the intersection points of the trajectory with the surface $(x_{1} + a)x_{3} = 1$.

Table 4 summarises the extremal values reached by $x_{1} = x_{3}$ and $x_{2}$ along a closed trajectory with an initial condition at $x(0) = (2.5,1.3,2.5)$.

**Table 4:** Presents $x_{j}$ extreme values corresponding to the middle axis $(\mu_{1} ,\mu_{2} ,\mu_{3} ) = (0,1,0)$ when $x_{1} (0) = x_{3} (0)$.

Item	Analytic statement	Estimated Values
General	$x_{1} = x_{3} ,$ $G_{3} = \frac{{x_{1}^{2} (0) + x_{2}^{2} (0)}}{2} + (a + b)x_{1} - \ln x_{1} (0).$	$x(0) = (2.5,1.3,2.5),$ $G_{3} = 3.9287.$
$x_{{1_{\max } }} ,$ $x_{{1_{\min } }}$	$x_{1} = x_{3} ,\,\,x_{2} = 0,$ $\frac{{x_{1}^{2} }}{2} + (a + b)x_{1} - \ln x_{1} = G_{3} .$	$x = (2.82,0,2.82),$ $x = (0.02,0,0.02).$
$x_{{2_{\max } }} ,$ $x_{{2_{\min } }}$	$x_{1} = x_{3} = 1,$ $x_{2} = \pm \sqrt {2[G_{3} - (a + b)] - 1} .$	$x = (1, \pm 2.48,\;1).$

### Unstable separatrix

For the case when $x_{1} (0) = - x_{3} (0)$, equations in (47) yield

80 $$x_{1} = b + x_{1} (0)e^{{ - \alpha_{3} }} ,\,\,\,\,\,\,\,\,\,x_{3} = x_{3} (0)e^{{ - \alpha_{3} }} .$$

Hence, equations in (50) produce

81 $$L = \frac{1}{2}[x_{1}^{2} (0) + x_{3}^{2} (0)] = x_{1}^{2} (0),\,\,\,\,\,\,\,\,\,M = x_{1} (0)x_{3} (0) = - x_{1}^{2} (0).$$

Therefore, $x_{1} = b + x_{3} ,\,\,L = - M,$ and then $N = 0$. Equation (51) results

82 $$\frac{{d^{2} \alpha_{3} }}{{d\tau^{2} }} = 1 - Le^{{ - 2\alpha_{3} }} + (a + b)x_{1} (0)e^{{ - \alpha_{3} }} .$$

It is evident that in this scenario, there are no EPs. A closer look at Eq. (81) shows that

83 $$\mathop {\lim }\limits_{\tau \to \infty } \frac{{d^{2} \phi_{2} }}{{d\tau^{2} }} = 1 \Rightarrow \mathop {\lim }\limits_{\tau \to \infty } x_{2} = \infty \Rightarrow \mathop {\lim }\limits_{\tau \to \infty } \alpha_{3} = \infty .$$

Utilizing Eqs. (80) and (81) in order to achieve

84 $$\mathop {\lim }\limits_{\tau \to \,\infty } x_{1} = \mathop {\lim }\limits_{\tau \to \,\infty } x_{3} = 0.$$

Therefore, a movement that quickly transitions into a pure spin in an upward direction around the central axis, while maintaining a constant angular acceleration is obtained.

## Discussion

The investigation delves into the influence of the GM and CBFTs on the rotatory motion of an asymmetric RB, employing Euler's dynamic equations to derive the governing EOM. To reduce reliance on inertia characteristics, the equations are appropriately scaled. By identifying and expanding the controlling EOM, inertia properties are effectively removed from the equation. The EPs of the dimensionless system are determined, alongside the linearized EOM, characteristic equation, and stability properties.

Three distinct cases are presented:

1. For applied constant torque on the minor axis, an analytic solution is achieved, complemented by a comprehensive diagrammed simulation illustrating SS, trajectories, stability zones, and extreme torque values in the 3D phase plane.
2. When considering a directed CBFT along the major axis, numerical solutions are provided, along with 3D and 2D graphs depicting dimensionless angular velocities leading to a typical spin-up maneuver. Motion stability varies with increasing GM values, while maintaining the spin-up maneuver about one of the dimensionless axes.
3. In the scenario of CBFT along the middle axis, an analytic solution is obtained, accompanied by complete 3D and 2D phase plane representations for various SS motions. Extreme value cases are explored, and stabilization is discussed in detail, including system solutions and tables of extreme values in the stable separatrix section.

The effects of different GM values on body paths and stabilization are thoroughly examined, yielding beneficial insights. Each instance undergoes a comprehensive assessment, analyzing obtained values at the lowest and highest points of angular velocity distinct dimensionless components along a periodic solution.

## Conclusion

The influence of the GM and CBFTs on the rotatory motion of an asymmetric RB is investigated. The governing EOM has been derived using Euler's dynamic equations and scaled to reduce their dependence on inertia characteristics. To remove their reliance on inertia properties, the controlling EOM has been identified and expanded. The EPs of the dimensionless system are determined, as well as the linearized EOM, characteristic equation, and stability properties. In three distinct cases.

The remarkable significance of these results becomes apparent when considering the advancement and evaluation of systems that utilize asymmetric RBs, such as satellites and spacecraft. The study's findings hold particular promise for stabilizing spacecraft motion within their orbits. Manipulating external moment values and body parameters can further enhance stabilization efforts. Consequently, these results may drive further exploration of GM perspectives in similar scenarios, with potential impacts across diverse industries, including engineering and astrophysics applications.

## Data Availability

Data sharing not applicable to this article as no datasets were generated or analyzed during the current study.
